# HLA alleles and sustained peanut consumption promote IgG4 responses in subjects protected from peanut allergy

**DOI:** 10.1172/JCI152070

**Published:** 2022-01-04

**Authors:** Kanika Kanchan, Stepan Grinek, Henry T. Bahnson, Ingo Ruczinski, Gautam Shankar, David Larson, George Du Toit, Kathleen C. Barnes, Hugh A. Sampson, Mayte Suarez-Farinas, Gideon Lack, Gerald T. Nepom, Karen Cerosaletti, Rasika A. Mathias

**Affiliations:** 1 Division of Allergy and Clinical Immunology, Department of Medicine, School of Medicine, Johns Hopkins University, Baltimore, Maryland, USA.; 2 Department of Genetics and Genomic Sciences, Icahn School of Medicine at Mount Sinai, New York, New York, USA.; 3 Benaroya Research Institute at Virginia Mason, Seattle, Washington, USA.; 4 The Immune Tolerance Network, Bethesda, Maryland, USA.; 5 Department of Biostatistics, Bloomberg School of Public Health, Johns Hopkins University, Baltimore, Maryland, USA.; 6 The Department of Pediatric Allergy, Division of Asthma, Allergy and Lung Biology, King’s College London, and Guy’s and St. Thomas’ NHS Foundation Trust, London, United Kingdom.; 7 The Department of Medicine, University of Colorado, Anschutz, Colorado, USA.; 8 Department of Pediatrics, Allergy and Immunology and; 9 Department of Population Health Science and Policy, Icahn School of Medicine at Mount Sinai, New York, New York, USA.

**Keywords:** Genetics, Immunology, Genetic variation, MHC class 1, MHC class 2

## Abstract

We investigated the interplay between genetics and oral peanut protein exposure in the determination of the immunological response to peanut using the targeted intervention in the LEAP clinical trial. We identified an association between peanut-specific IgG4 and HLA-DQA1*01:02 that was only observed in the presence of sustained oral peanut protein exposure. The association between IgG4 and HLA-DQA1*01:02 was driven by IgG4 specific for the Ara h 2 component. Once peanut consumption ceased, the association between IgG4-specific Ara h 2 and HLA-DQA1*01:02 was attenuated. The association was validated by observing expanded IgG4-specific epitopes in people who carried HLA-DQA1*01:02. Notably, we confirmed the previously reported associations with HLA-DQA1*01:02 and peanut allergy risk in the absence of oral peanut protein exposure. Interaction between HLA and presence or absence of exposure to peanut in an allergen- and epitope-specific manner implicates a mechanism of antigen recognition that is fundamental to driving immune responses related to allergy risk or protection.

## Graphical abstract

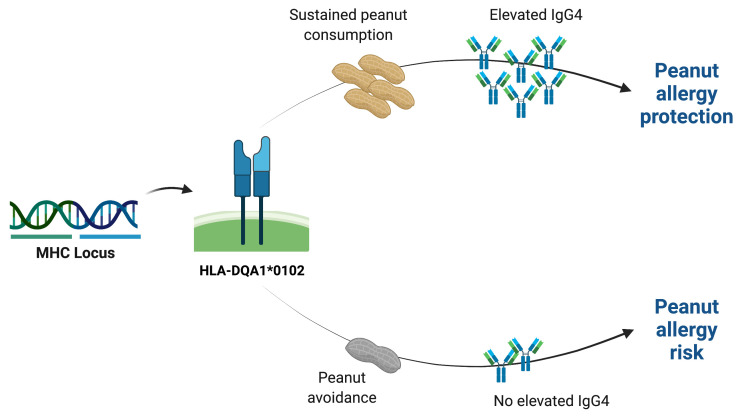

## Introduction

The interplay between an individual’s genetics and exposure to particular environmental or therapeutic modulators influences mechanisms of disease, prognosis, and treatment. Clinical trials of food allergy tolerance, in which controlled exposure to particular allergens is used for immunotherapy, present a unique opportunity for interrogating the interaction between a targeted intervention and specific genotypes, enabling exploration of the potential for personalized therapy.

Food allergy, like many immunological disorders, is associated with multiple genes, most of which have small individual contributions to expression of the allergic clinical phenotype (1). Notably, genetic associations with specific HLA polymorphisms have been identified in association with allergy to peanuts (2, 3); these associations appear to be specific to peanut and not to other allergenic foods (2–4). HLA genes have been extensively studied in the context of autoimmune diseases, in which HLA polymorphisms are disease specific and consistent with the primary known function of HLA molecules, i.e., presenting peptide antigens for immune recognition (5).

Learning Early About Peanut Allergy (LEAP) was a prospective, randomized, clinical trial of young children at high risk of peanut allergy (PA) conducted at a single site in the United Kingdom (6). The participants enrolled in the LEAP trial were at least 4 months and less than 11 months of age and had severe eczema, egg allergy, or both. Five years of controlled ingestion of dietary peanut protein in the LEAP trial resulted in prevention of subsequent PA (6). The prevalence of PA at 60 months of age in the LEAP cohort (*n* = 628) was 17.2% in the avoidance group and 3.2% in the consumption group, and results were even stronger in the per-protocol population. This trial led to the adoption of clinical guidelines for early introduction of peanut into the diet as a safe and effective intervention (7). We performed whole genome sequencing (WGS) on the subjects in the LEAP trial and have previously reported an association with progression to PA in the avoidance arm of this study, where *MALT1* genotypes distinguished children who developed PA by 5 years of age from nonallergic participants (8).

In the peanut-consumption arm of the study, the immunological response to peanut was characterized by an elevation in peanut-specific IgG4 (psIgG4) levels. We now report the identification of a genotype associated with psIgG4 in participants who consumed peanut during the LEAP study, localized to the HLA region on chromosome 6p21. Remarkably, this association identified a specific HLA class II allele, DQA1*01:02, which is the same allele previously implicated in susceptibility to PA in population studies (2, 3) with unmeasured environmental exposure. Whereas *MALT1* genotypes correlated with broad immunological indicators of PA that were not restricted to individual allergenic components of peanut protein, this HLA-DQA1*01:02 association with psIgG4 was predominantly correlated with a single protein component, Ara h 2. The elevation of psIgG4 was dependent on the combination of this specific genotype and consumption of peanut, indicating a gene-environment interaction effect that is consistent with the preferential ability of immune responses in children with this HLA class II genotype to recognize specific peanut-allergen peptides.

## Results

### Association of HLA alleles and haplotypes with primary phenotypes in the context of peanut exposure.

To identify HLA genetic determinants of PA and quantitative traits of psIgG4 and psIgE, study participants were stratified on the basis of the intervention (i.e., avoidance vs. consumption of peanut), and we tested for associations between HLA alleles and haplotypes and 3 primary phenotypes, including psIgG4, psIgE, and PA, at 60 months. psIgG4 and psIgE association tests were performed in both strata, while association tests for PA were limited to the avoidance group, given that only 1 participant was peanut allergic in the consumption group (Table 1). HLA class I and class II alleles were imputed from WGS data, and genes with a call rate of less than 95% were filtered out. Analysis was limited to 38 alleles with a frequency of 5% or more for each passing gene. The full set of association results for each of the 38 imputed HLA alleles meeting these criteria at 8 HLA genes and 3 primary phenotypes is shown in Supplemental Table 1 (supplemental material available online with this article; https://doi.org/10.1172/JCI152070DS1). We identified 10 HLA alleles with *P* < 0.05 for any 1 of the 3 primary phenotypes (Table 2). The only allele to meet the threshold of significance (*P* < 0.001) to correct for the multiple testing across 38 alleles was HLA-DQA1*01:02 with psIgG4 (*P* = 2.21 × 10^–4^) in the consumption group. Even with a more stringent correction for 3 phenotypes tested, this association retained its significance (*P* < 0.0004).

It is important to highlight that the very same HLA class II DQA1*01:02 allele associated with higher psIgG4 in the consumption group was nominally associated with increased risk for PA in the avoidance group (OR = 1.99, *P* = 4.37 × 10^–2^). In fact, this specific allele has been previously implicated for PA risk (OR = 1.81, *P* = 1.75 × 10^–2^) (3) in population-based case-control studies. To confirm that this association with PA does not confound the associations noted with psIgG4, we reran our association tests by adjusting for PA and by limiting the analysis to only those participants that were not peanut allergic. We found our results to remain the same (Supplemental Table 2); there was no association with psIgG4 in the avoiders even when PA was considered, and given only a single participant with PA in the consumers, there was no change in this group.

Given that HLA alleles are recognized as having population variability and potentially even as population specific, we restricted our analysis to those participants with self-reported European ethnicity (Supplemental Table 3). The associations with psIgG4 (*P* = 3.34 × 10^–4^) remained largely unchanged from the analysis based on the full group above, both in statistical significance and effect size. The associations between HLA-DQA1*01:02 allele and PA risk were stronger in the group of self-reported European participants (OR = 3.54, *P* = 3.63 × 10^–3^) compared with that noted in the full group of LEAP participants. None of the associations in the smaller non-European groups were significant given limitations in power.

The allelic associations with psIgG4 in the consumption group are largely represented by the haplotypes across the *HLA-DQA1* and *HLA-DQB1* class II genes (Table 2 and Supplemental Table 4). We found significant associations for 2 haplotypes: HLA-DQA1*01:02~HLA-DQB1*06:02 (*P* = 2.69 × 10^–2^) and HLA-DQA1*05:01~HLA-DQB1*02:01 (*P* = 4.31 × 10^–3^). The haplotype HLA-DQA1*01:02~HLA-DQB1*06:02 had a less significant result relative to the HLA-DQA1*01:02 allele (*P* = 2.69 × 10^–2^ vs. *P* = 2.21 × 10^–4^, respectively), but this is likely due to reduced power, given the lower frequency of the haplotype compared with the allele (11% vs. 17%, respectively) in the LEAP study participants.

### Association of HLA-DQA1*01:02 with IgG4 to peanut components.

**Figure 1:**
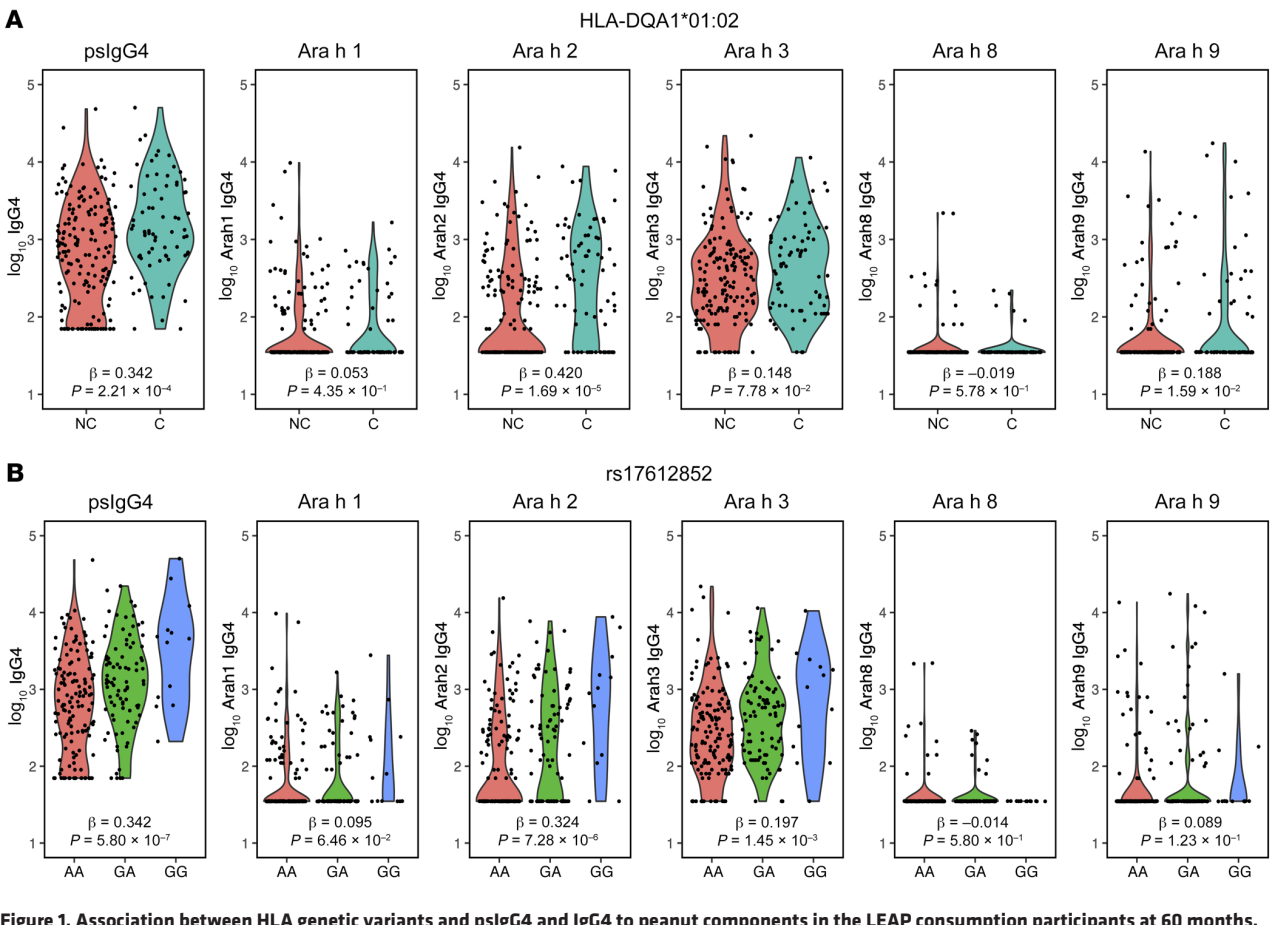
Association between HLA genetic variants and psIgG4 and IgG4 to peanut components in the LEAP consumption participants at 60 months. (**A**) Allele HLA-DQA1*01:02 was analyzed under a dominant model. NC, noncarriers; C, carriers of either 1 or 2 copies of HLA-DQA1*01:02 allele. (**B**) SNP rs17612852 was analyzed under the additive model for the genotypes AA/GA/GG coded as 0/1/2 in a linear model. The effect sizes and *P* values from the linear regression model are shown. *n* = 267.

Given the importance of HLA class II molecules in antigen presentation, we tested specifically for HLA-DQA1*01:02 association with levels of IgG4 to 5 individual peanut components (Ara h 1, h 2, h 3, h 8, and h 9) in the LEAP consumption group at 60 months (Figure 1A). We determined the only statistically significant association (after correction for multiple testing) to be Ara h 2–specific IgG4 (*P* = 1.69 × 10^–5^); none of the other peanut component–specific IgG4 levels at 60 months were associated with HLA-DQA1*01:02 (*P* > 0.05) after multiple comparisons correction. In fact, this association with Ara h 2 was stronger than the association noted for (total) psIgG4 (β = 0.420, *P* = 1.69 × 10^–5^ vs. β = 0.342, *P* = 2.21 × 10^–4^, respectively) and suggests a component-specific effect of the HLA allele (Figure 1A) where it largely drives IgG4 to Ara h 2 at 60 months with little to no effect on Ara h 1, h 3, h 8, and h 9.

### Association of SNPs mapping to the HLA locus and primary phenotypes in the context of peanut exposure.

Given the availability of GWAS genotype data and prior evidence that SNPs mapping to the HLA locus are determinants of PA, in addition to HLA alleles, we tested for associations between 9742 SNPs mapping to the HLA region (Chr6:28,477,797-33,448,354/GRCh37) and 3 primary phenotypes, including psIgG4, psIgE, and PA, at 60 months (Supplemental Figure 1). Similarly to what occurred with the HLA alleles above, psIgG4 and psIgE association tests were performed in both strata; for PA, association tests were limited to the avoidance group only. At a threshold of *P* < 5.13 × 10^–6^, the threshold to account for testing 9742 SNPs, only a single intergenic SNP (rs17612852) between the *HLA-DQA1* and *HLA-DQB1* genes showed significant association (*P* = 5.80 × 10^–7^) with psIgG4 in the peanut-consumption group (Supplemental Figure 1). As shown in Supplemental Figure 2, conditional analyses on this peak variant (rs17612852) confirmed the presence of only a single regional association (i.e., the observed association in panel A is no longer present in panel B when conditioned on rs17612852). No further associations were observed at *P* < 5.13 × 10^–6^ for psIgG4 in the peanut-avoidance group, psIgE in either group, or PA in the avoidance group (Supplemental Figure 1) at 60 months.

Similarly to HLA-DQA1*01:02, rs17612852 was also tested for its association with levels of IgG4 to 5 individual peanut components in the LEAP consumption group (Figure 1B) at 60 months. We found that the association with Ara h 2–specific IgG4 (*P* = 7.28 × 10^–6^) was significant at 60 months. In contrast to HLA-DQA1*01:02, the association of rs17612852 with Ara h 2 was weaker than the association noted for psIgG4 (β = 0.324, *P* = 7.28 × 10^–6^ vs. β = 0.342, *P* = 5.80 × 10^–7^; Figure 1B) at 60 months, and association was also noted for Ara h 3 (*P* = 1.45 × 10^–3^).

There is well-documented linkage disequilibrium (LD) across variants in the HLA genes, and it is difficult to tease apart the effects of SNPs versus specific HLA alleles. There is strong LD (D′ = 0.99, *r^2^* = 0.65) between rs17612852 and HLA-DQA1*01:02, and considering also the small sample size of the LEAP consumption group (*n* = 267), we were unable to further separate the independence of the association between the peak SNP and the allele for Ara h 2 IgG4 with statistical modeling.

### Effect of rs17612852 and HLA-DQA1*01:02 on HLA-DQB1 RNA.

**Figure 2:**
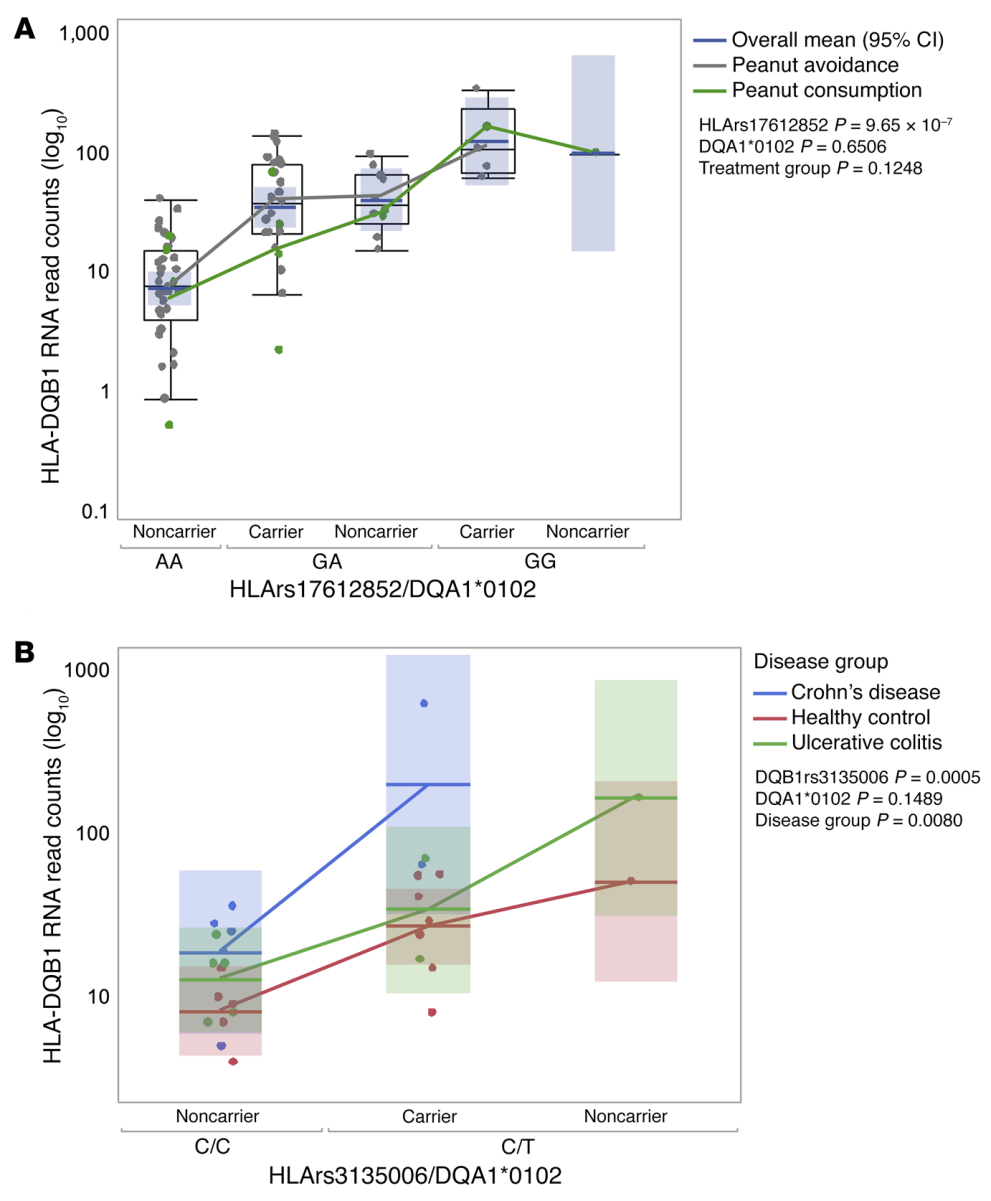
Association among HLA SNP rs17612852, allele HLA-DQA1*01:02 carrier status, and *HLA-DQB1* RNA expression. (**A**) DQB1 RNA read counts from bulk RNA-Seq of CD4^+^ T cells from LEAP trial participants (*n* = 86). Blue horizontal lines and blue shaded areas show the overall means with 95% CI. Gray dots represent the peanut-avoidance group (*n* = 63) and green the peanut-consumption group (*n* = 23) with lines connecting respective group means. (**B**) Colon biopsies (*n* = 28) from uninflamed tissue of patients with Crohn’s disease, healthy controls, and patients with ulcerative colitis. Horizontal lines and shaded areas in the box plots show the means with 95% CI for the disease group, and means are connected with colored lines. Mutually adjusted type 3 *P* values were reported for each term in the models.

The HLA SNP, rs17612852, is a significant cis-expression quantitative trait locus (cis-eQTL) for 24 genes in gut tissues and whole blood in GTEx and eQTLGen expression data sets (*P* values < 1 × 10^–5^). Here, we tested to determine whether HLA-DQA1*01:02 carrier status and rs17612852 were eQTLs for expression of 7 HLA class II genes in bulk RNA-Seq data from CD4^+^ T cells isolated from PBMCs of 86 LEAP trial participants in both the avoidance and consumption groups. Normalized read counts from the *HLA-DQA1* and *HLA-DQB1* genes were associated with carrier status at DQA1*01:02 and genotype at rs17612852 (Supplemental Table 5). A significant increase in *HLA-DQB1* RNA levels was detected in CD4^+^ T cells with each copy of the rs17612852 minor allele (*P* = 8.34 × 10^–13^) or DQA1*01:02 carrier status (*P* = 1.27 × 10^–5^), mirroring the allelic effect on psIgG4 levels. *HLA-DQA1* RNA levels were also significantly associated with SNP genotype (*P* = 1.66 × 10^–7^) and DQA1 carrier status (*P* = 7.72 × 10^–4^). In a multivariate analysis, we found that the SNP rs17612852 was the primary determinant of *HLA-DQB1* expression (*P* = 7.04 × 10^–7^; Figure 2A), and neither DQA1*01:02 carrier status nor treatment group provided additional contributions to the association between rs17612852 and *HLA-DQB1* expression (*P* = 0.6506 and *P* = 0.1248 for dominant effects, respectively).

More relevant to oral exposure, we obtained similar results using bulk RNA-Seq data from gut tissue. *HLA-DQB1* normalized read counts from uninflamed colon biopsies (*n* = 28) from healthy control subjects or patients with Crohn’s disease or ulcerative colitis were stratified according to DQA1*01:02 carrier status and genotype at the SNP rs3135006, which is in LD with rs17612852 in the LEAP cohort (D′ = 0.98, *r^2^* = 0.79). Multivariate analysis showed a significant increase in *HLA-DQB1* RNA levels with the minor allele at rs3135006 (*P* = 5 × 10^–4^), but not the DQA1*01:02 allele (*P* = 0.1489; Figure 2B). These results support the hypothesis that the eQTL represented by rs17612852/rs3135006 SNPs influences class II gene expression primarily at the *HLA-DQB1* gene, with significantly less impact on expression on the *HLA-DR* and *HLA-DP* class II genes (Supplemental Table 5).

### Fine mapping of HLA class II region and colocalization between HLA class II eQTLs and phenotype association.

For genetic fine mapping, we used the results of 1908 SNPs (Chr6:32,100,000–32,800,000) mapping to the HLA class II region interpolated with 14 class II alleles for psIgG4 and 7 HLA class II genes from the CD4^+^ T cells of 86 LEAP trial participants (Supplemental Figure 3). At the regional level, significant eQTLs were only identified for 2 of the 7 genes (*HLA-DQA1* and *HLA-DQB1*; Supplemental Figure 3). While rs17612852 was significant for both genes (Supplemental Table 5 and Supplemental Figure 3), it was the peak association for *HLA-DQB1*, but not *HLA-DQA1* (i.e., other stronger eQTLs were noted for *HLA-DQA1* expression). This alignment of the peak associated variant between the trait of interest (psIgG4) and gene expression is further illustrated in Supplemental Figure 4A; perfect alignment is noted for *HLA-DQB1*, but not *HLA-DQA1* eQTLs. SuSie was run to identify credible sets of potentially causal variants for psIgG4, Ara h 2–specific IgG4, *HLA-DQA1*, and *HLA-DQB1* (Supplemental Figure 3). Credible sets were only identified for *HLA-DQB1* at a coverage of greater than 0.95; 1 credible set was identified, and it consisted of only rs17612852 with a posterior inclusion probability (PIP) of 0.99 (Supplemental Figure 4B). Given small sample sizes, no resolution of credible sets was possible for psIgG4, Ara h 2, and *HLA-DQA1* at a coverage of 0.95. Finally, the perfect alignment of peak associations between psIgG4 and eQTLs for *HLA-DQB1* expression was confirmed by Bayesian colocalization where the PP4 for rs176128652 was exactly 1 between the 2.

### Associations between HLA and MALT1 with psIgE and psIgG4 in the context of peanut exposure.

In a previous GWAS on LEAP participants, SNP rs57265082 on chromosome 18, mapping to the mucosa-associated lymphoid tissue lymphoma translocation (*MALT1*) gene was identified as associated with PA in the peanut-avoidance group (*P* = 6.49 × 10^–8^; ref. 8). It was also found to be significantly associated with the number of psIgE components to Ara h 1, h 2, and h 3 at 60 months (8). Here, we tested for temporal patterns in the associations for HLA and *MALT1* within the 2 intervention strata. Specifically, we evaluated the associations of HLA-DQA1*01:02 and rs57265082 at baseline, 12, 30, and 60 months in the 2 strata with psIgG4 and IgG4 to peanut components Ara h 1, h 2, h 3, h 8, and h 9 or psIgE and IgE to these peanut components. The numbers of participants included in the temporal pattern analyses for each phenotype are shown in Supplemental Table 6.

Three points are in evidence from these analyses. First, looking at each time point modeled individually (Supplemental Figure 5), in the consumption group, HLA-DQA1*01:02 was strongly associated with psIgG4 levels, but not psIgE (Supplemental Figure 5A). In contrast, in the avoidance group, *MALT1* was associated with both psIgE and psIgG4, but the association was to a greater degree for psIgE than psIgG4 (Supplemental Figure 5B). In multivariant modeling, the *MALT1* association with psIgG4 in the avoidance group (Supplemental Figure 5B) was found to be largely due to the association between *MALT1* and psIgE (Supplemental Table 7) and not a primary/independent effect of *MALT1* on psIgG4; at 60 months, the association between *MALT1* and psIgG4 decreased from *P* = 0.0002 to *P* = 0.0312 once psIgE was included in the model within the avoidance group. In contrast, the association between HLA and psIgG4 remained unchanged when psIgE was included in the model in the consumption group, supporting a primary/independent effect of HLA-DQA1*01:02 on psIgG4.

Our second observation is that the associations between IgG4 phenotypes and HLA-DQA1*01:02 were not replicated in the avoidance group (Supplemental Figure 6A). Likewise, the associations between *MALT1* and IgE phenotypes were largely not replicated in the consumption group (Supplemental Figure 6B). This suggests that both HLA-DQA1*01:02 and rs57265082 have an environmental interaction, and this is confirmed in a model including all subjects together at 60 months. As noted in Supplemental Table 7, all interactions with exposure were significant (*P* = 0.01 for peanut consumption*rs57265082; *P* = 0.0024 for peanut consumption*HLA-DQA1*01:02; asterisks represent “interaction” term in the model between the environment [consumption] and genetics [either rs57265082 or HLA-DQA1*01:02]).

Third, when examining the effects of these 2 genetic loci beyond total psIgE and psIgG4 and at the level of individual components at each individual time point, we noted that HLA-DQA1*01:02 largely determined Ara h 2 IgG4 (Supplemental Figure 5A) and not Ara h 1, h 3, and h 8 representing a component-specific association; however, *MALT1* seemed to have almost equal effects across IgEs to multiple peanut components: Ara h 1, h 2, and h 3 (Supplemental Figure 5B), representing a nonspecific association.

**Figure 3:**
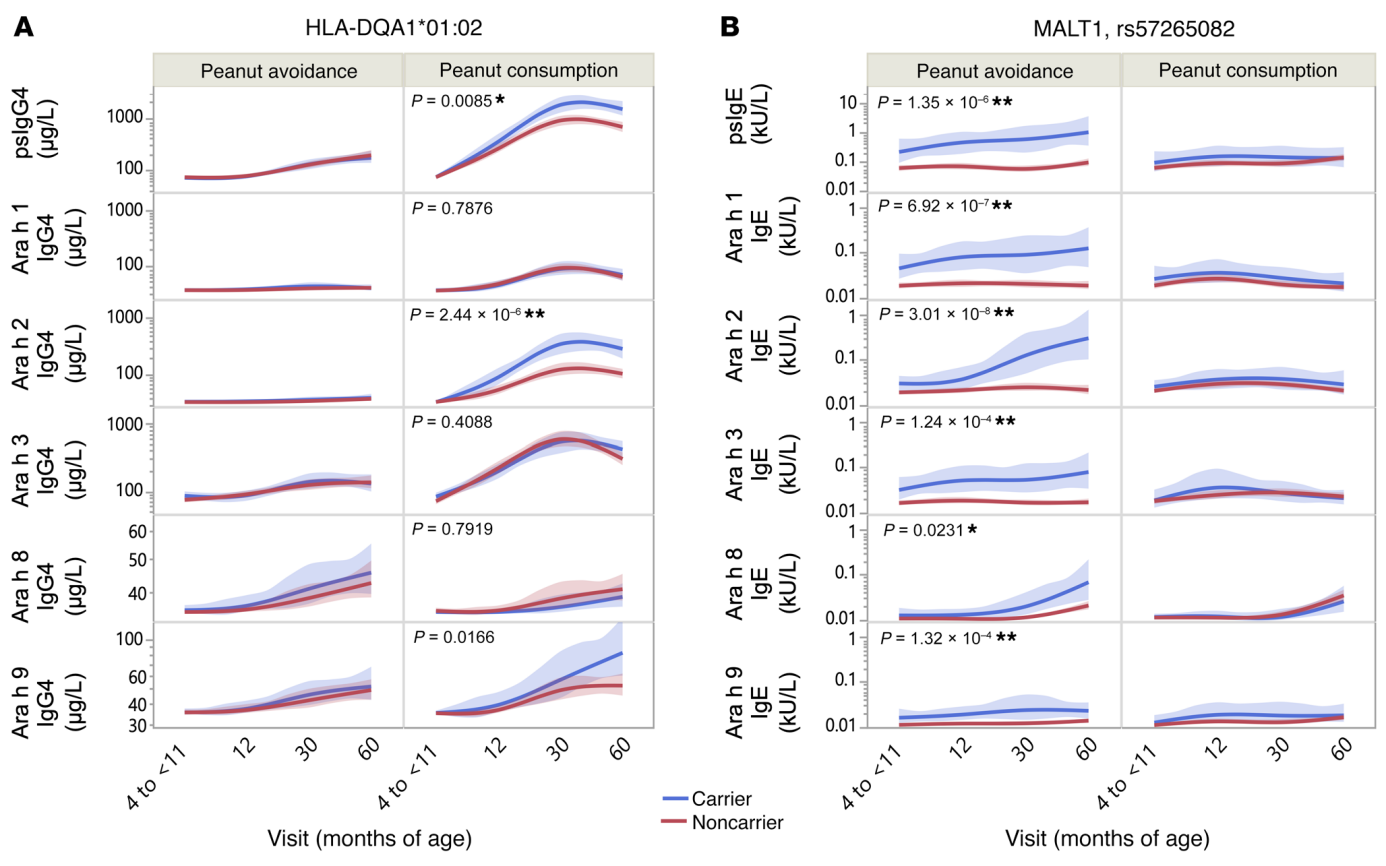
Relative distribution of psIgG4 and psIgE, and IgG4 and IgE to peanut components by carrier status. (**A**) HLA-DQA1*01:02 and (**B**) *MALT1* SNP rs57265082. Unadjusted mean values of IgG4 phenotypes stratified by HLA-DQA1*01:02 carrier status and IgE phenotypes stratified by *MALT1* carrier status at each assessment and by treatment group assignment are shown with bootstrapped 95% CIs and *P* values. The associations between IgE and *MALT1* are robust to the baseline differences in IgE as previously demonstrated by Winters et al. (8). *P* values represented are those for the main effect of carrier status, and full results from the model are presented in Supplemental Table 8, A and B. *P < 0.05; **P < 0.0001. The peanut-avoidance and peanut-consumption groups included *n* = 275 and *n* = 267 participants, respectively.

We extended the individual time-point analyses above to a mixed effects linear model including all time points together and where each outcome was fit with predictors: treatment group, age at assessment, HLA-DQA1*01:02 carrier status, interaction effect between the carrier status and treatment, and interaction effect between ages at assessment and treatment (Figure 3 and Supplemental Table 8, A and B). These models confirm the following: (a) Ara h 2 IgG4 specificity of the HLA associations (Figure 3A) in contrast with *MALT1* associations noted across Ara h 1, h 2, and h 3 for IgE (Figure 3B); and (b) the striking gene environment interactions with prominent differences between avoiders and consumers when modeling carriers and noncarriers at HLA-DQA1*01:02 and *MALT1*. The association between HLA-DQA1*01:02 and psIGg4 was significant (*P* = 8.5 × 10^–3^) and was specific for Ara h 2 IgG4 (*P* = 2.44 × 10^–6^) but not Ara h 1, h 3, h 8, or h 9 (Figure 3A). Additionally, while the interaction effect between HLA-DQA1*01:02 and the treatment group was significant for psIgG4 (*P* = 2.3 × 10^–3^), it was considerably stronger for Ara h 2 IgG4 (*P* = 2.74 × 10^–5^). On the other hand, *MALT1* (Figure 3B) was significantly associated with all IgE phenotypes (psIgE, *P* = 1.35 × 10^–6^; Ara h 1, *P* = 6.92 × 10^–7^; Ara h 2, *P* = 3.01 × 10^–8^; Ara h 3, *P* = 1.24x10^–4^; Ara h 8, *P* = 2.31 × 10^–2^; Ara h 9, *P* = 1.32 × 10^–4^). Finally, the interaction effect between *MALT1* and the treatment group was significantly associated with all outcomes (psIgE, *P* = 4.55 × 10^–4^; Ara h 1, *P* = 0.0023; Ara h 2, *P* = 1.37 × 10^–4^; Ara h 3, *P* = 0.0013; Ara h 8, *P* = 0.0029) except that of Ara h 9 (*P* = 0.2180).

All LEAP participants were asked to cease consumption of peanuts for the period between 60 and 72 months of age, at which point in time psIgE and psIgG4 were once again assayed in a set of *n* = 478 (Supplemental Table 6) LEAP-ON participants (9). To determine whether the environmental interaction was sustained past the point of 60 months (i.e., the critical time point at which the consumers cease oral peanut consumption), the above analyses were extended to 72 months (Supplemental Figures 5, 6, and 7). Based on the individual time-point analyses (Supplemental Figures 5 and 6), associations were found to increase in statistical significance over time, with a general doubling in effect sizes going from 12 months to 30 months, the strongest association noted at 60 months, and a rapid decline in the strength of the association at 72 months. The association between HLA and IgG4 to Ara h 2 in the consumption group was nonsignificant (*P* = 0.139), and association with psIgG4 went down dramatically (*P* = 1.66 × 10^–2^). In contrast, for *MALT1* on IgE in the avoidance group, statistical significance remained strong (*P* < 10^–4^). It should be pointed out that there was a drop in sample size between the 60-month and 72-month visits as outlined in Supplemental Table 6 in both the avoider and consumer groups for both IgG4 and IgE. The patterns noted in the individual time-point analyses are made clear in the full linear mixed models presented in Supplemental Figure 7, where the carrier and noncarrier lines converge at 72 months for IgG4 by HLA-DQA1*01:02 carrier status, but not for IgE by *MALT1*.

### Selective epitope expansion of IgE and IgG4 to specific peptides by MALT1 (rs57265082) and HLA-DQA1*01:02 carrier status, respectively.

Five-year changes in sequential-epitope–specific IgE (ses-IgE) and IgG4 (ses-IgG4) profiles of the LEAP trial participants were compared in a subset of LEAP avoiders and consumers by outcome at 60 months (Supplemental Table 9). The strongest expansion for ses-IgG4 by HLA-DQA1*01:02 genotype was noted in the sensitized subgroup of consumers (*n* = 94) (Supplemental Figure 8A) and for ses-IgE by the *MALT1* (rs57265082) genotype in the allergic subgroup of avoiders (*n* = 38; Supplemental Figure 8B). Therefore, subsequent testing by carrier status at HLA-DQA*01:02 and *MALT1* (rs57265082) was limited to the sensitized subgroup of consumers for ses-IgG4 and the allergic subgroup of avoiders for ses-IgE, respectively.

**Figure 4:**
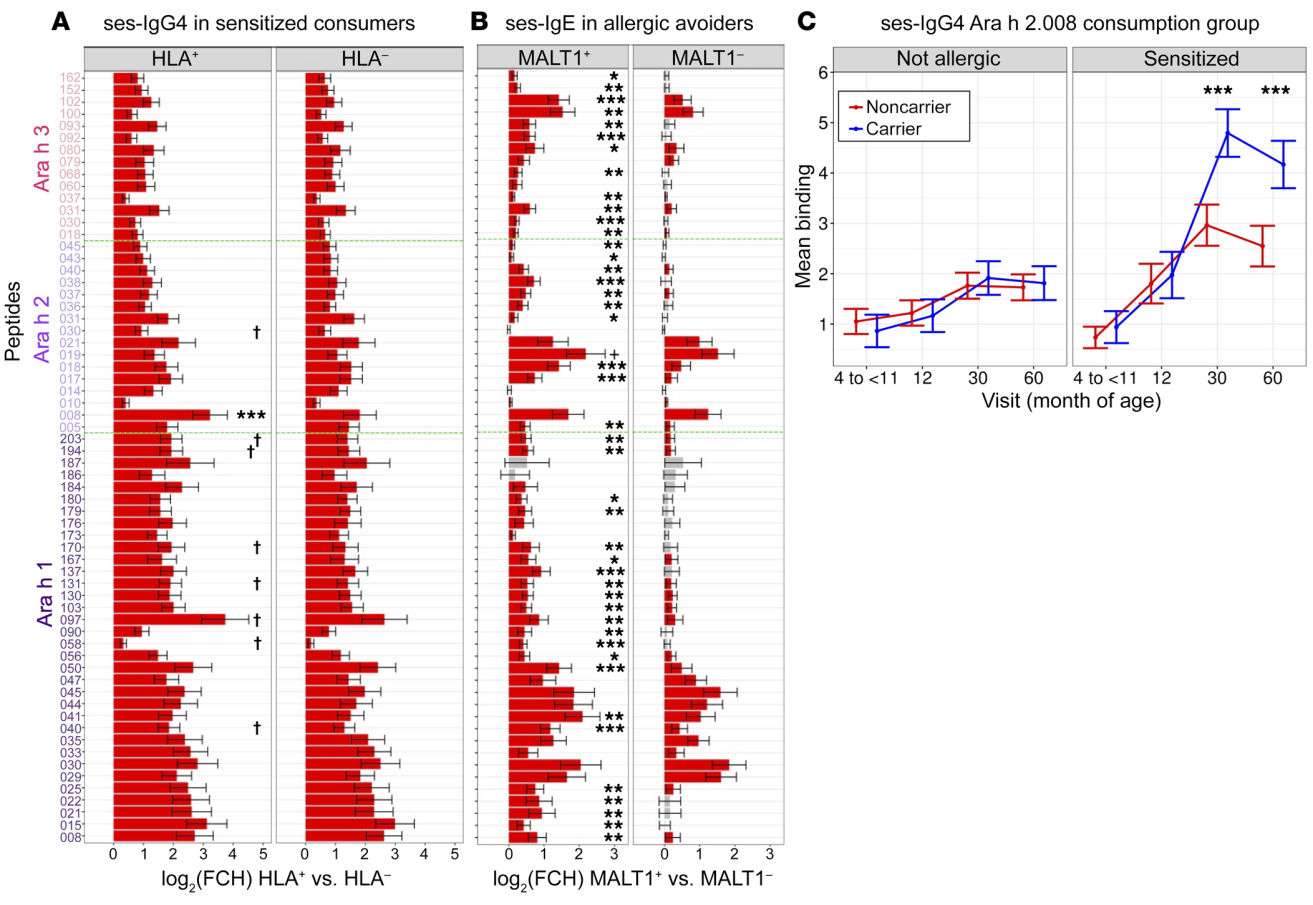
Expansion of linear epitopes in ses-IgG4 and ses-IgE at 60 months versus baseline visit. (**A** and **B**) Bars represent the changes in binding from baseline to 60-month visit in log_2_ fold change. Red indicates significant expansion (FDR < 0.05); asterisks indicate significance in differences between the 2 genotypes, carrier (HLA^+^ and MALT1^+^ panels) and noncarrier (HLA^–^ and MALT1^–^ panels): *FDR < 0.05; **FDR < 0.01; ***FDR < 0.001; ^†^FDR < 0.1. (**A**) ses-IgG4 expansion by HLA-DQA1*01:02 genotype among children (*n* = 161) in the peanut-consumption group who became sensitized by visits at 60 months. (**B**) ses-IgE expansion by *MALT1* genotype status in children enrolled in the avoidance group (*n* = 162) who developed PA after 60 months and (**C**) ses-IgG4 Ara h 2.008 epitope expansion by HLA-DQA1*01:02 in sensitized versus nonsensitized, nonallergic subjects in the consumption group (carriers, blue; noncarriers, red); asterisks indicate significance in differences between the 2 genotypes as in **A** and **B**. Error bars in all panels correspond to CIs. Data are represented as mean ± 95%CI.

In comparing 60 months to baseline, 9 epitopes had significantly (*P* < 0.05, FDR < 0.1) stronger ses-IgG4 expansion in HLA-DQA*01:02 carriers than noncarriers (Figure 4A); however, only 1 association had an FDR < 0.001; Ara h 2.008 epitope expansion was noticeably stronger compared with the other 8. In contrast, the differences in epitope expansion for *MALT1* carriers versus noncarriers were strong and nearly ubiquitous across all epitopes for Ara h 1, h 2, and h 3 (Figure 4B). An examination of the temporal changes in Ara h 2.008 epitope expansion by HLA carriers versus noncarriers (Figure 4C) extends this striking difference in expansion between the carriers and noncarriers over time from baseline to 30 and 60 months, and it was noted only in sensitized participants.

**Figure 5:**
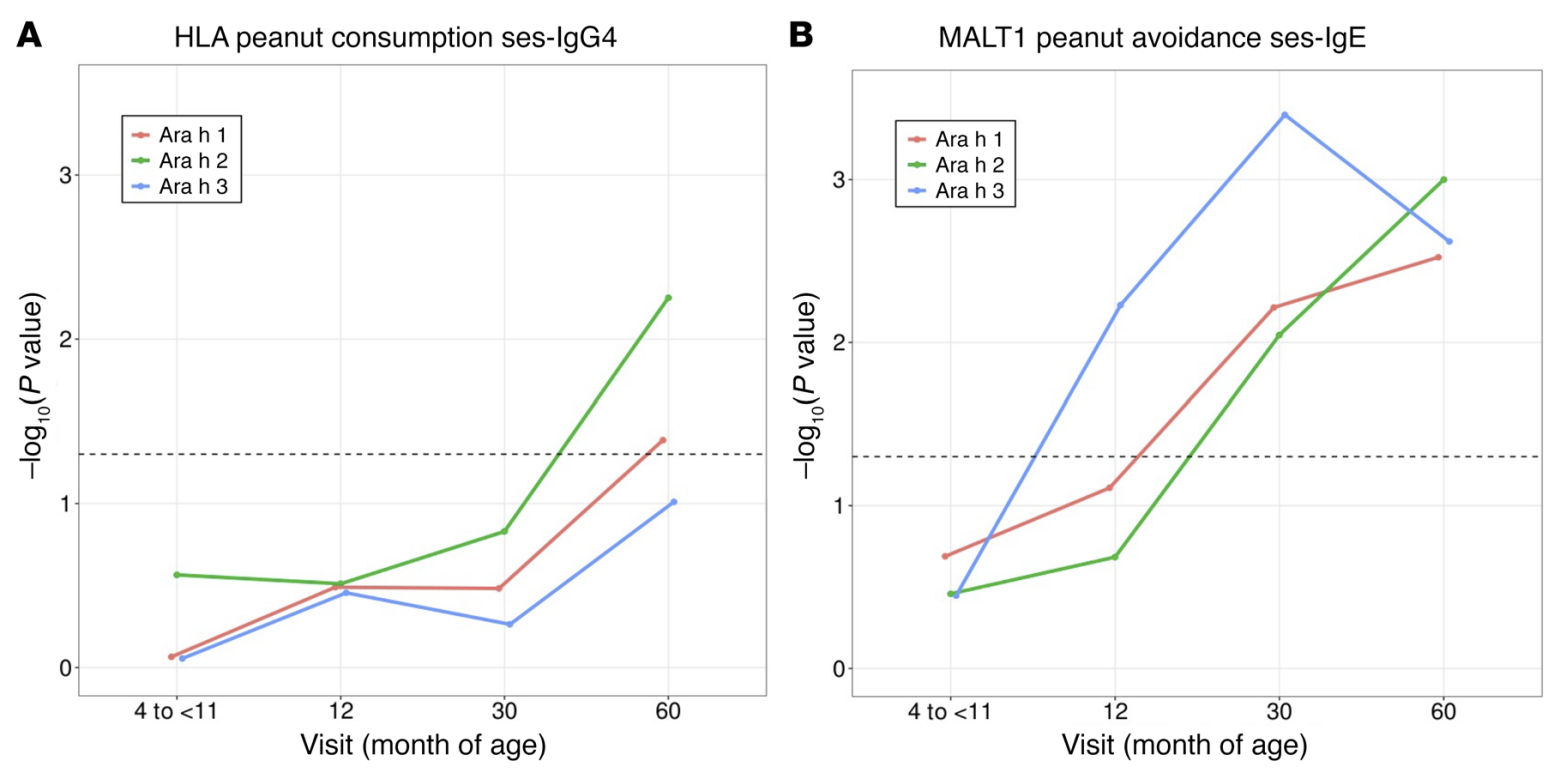
CCA evaluating the association of the linear epitopes overall ses-IgG4 and ses-IgE binding to Ara h 1, Ara h 2, and Ara h 3 with HLA-DQA1*01:02 and *MALT1* rs57265082, respectively. (**A**) Permutation *P* values of ses-IgG4 linear epitopes’ association with HLA-DQA1*01:02 in participants from the consumption group (*n* = 161). (**B**) Permutation *P* values of ses-IgE linear epitopes association with *MALT1* in the avoidance group (*n* = 162). Dashed lines indicate significance levels at *P* = 0.05.

In order to evaluate whether the component-specific association with genetic variants is also observed at the sequential epitope level, we performed canonical correlation analysis (CCA) evaluating the multivariate association of the sequential epitopes sesIgG4 and sesIgE binding to Ara h 1, Ara h 2, and Ara h 3. This analysis shows that for HLA-DQA*01:02 (Figure 5A), significance was largely noted for Ara h 2 at 60 months (*P* = 6 × 10^–3^), but for *MALT1* rs57265082 (Figure 5B), significance was noted across Ara h 1 at 30 and 60 months (*P* = 6 × 10^–3^ and *P* = 3 × 10^–3^, respectively), Ara h 2 at 30 and 60 months (*P* = 9 × 10^–3^ and *P* = 1 × 10^–3^, respectively), and Ara h 3 at 12, 30, and 60 months(*P* = 6 × 10^–3^, *P* = 4 × 10^–4^ and *P* = 2 × 10^–3^, respectively). We also confirmed that the associations were limited to HLA-DQA*01:02 and ses-IgG4 in the consumption (and not avoidance, Supplemental Figure 9A) group and those for *MALT1* and ses-IgE were limited to the avoidance (and not consumption, Supplemental Figure 9B) group. These data validate the associations noted between the 2 genetic variants and psIgE and psIgG4 in the context of environmental exposure and specificity of response to components.

### HLA genetic associations with psIgG, IL-4, and IL-10 in LEAP consumers.

In an attempt to determine whether there were residual associations with IgG subtypes beyond IgG4, we expanded the analyses to total psIgG. First, we did indeed observe associations for psIgG (Supplemental Table 10) in the consumption group in LEAP. We noted that the associations with psIgG were less significant and had lower effect sizes than those noted for psIgG4. Second, we observed that any association between the HLA variants and psIgG went away fully when we adjusted for either total psIgG4 or component-specific Ara h 2 IgG4 (*P* values were fully nonsignificant, and the effect sizes were vastly reduced). Our conclusion from these models is that the primary IgG subtype with respect to the HLA associations are for IgG4. We performed analysis in the LEAP consumers to understand whether the HLA associations observed for IgG4 were parallel for circulating IL-4 and IL-10 at the 60-month visit. We did not observe any genetic associations between the cytokines and genetic variants (*P* > 0.5, data not shown). Second, we looked at the correlations between the cytokines (IL-10, IL-4, and ratio of IL-10/IL-4) and psIgG4 and psIgE directly and we did not observe any direct correlation between circulating cytokine levels and immunoglobulins.

## Discussion

Immune responses to environmental antigens, such as the allergenic peanut proteins administered in the LEAP prevention study, present an opportunity for interrogating the specific role genes play in an individual’s response to controlled antigen exposure. Elevation of psIgG4 antibody in participants who consumed peanut in LEAP (6) represents such a response; in the trial arm in which peanut consumption was avoided for the 5-year duration of this study, psIgG4 was much lower than in the peanut-consumption arm and the rate of PA was much higher. The specific role of peanut exposure–induced IgG4 in the prevention of PA is not known. In therapeutic allergen immunotherapy, IgG4 is believed to act as a blocking antibody that takes up allergen and prevents its interaction with IgE on the surface of mast cells and basophils. For example, depletion of IgG4 from the plasma of peanut-allergic individuals undergoing oral immunotherapy with peanut restores the ability of peanut allergen to induce mast cell activation in vitro (10). It is conceivable that, when allergen is introduced early as a method of prevention of food allergy, the same blocking mechanism of IgG4 contributes to reduced IgE-antigen crosslinking, reduced type 2 cytokine production by CD4^+^ T cells, and reduced production of IgE, thus facilitating a state of tolerance that persists even when IgG4 levels decline after prolonged consumption or cessation of antigen exposure, as seen at 72 months in the LEAP trial. However, this hypothesis has not been tested. Here, we found that levels of psIgG4 in LEAP participants who were randomized to consume peanut were strongly associated with HLA variant represented by the HLA-DQA1*01:02 allele in the HLA class II region on chromosome 6 as well as with a closely linked SNP marker that is associated with levels of expression of the HLA-DQ (*HLA-DQB1* and *HLA-DQA1*) molecules. This association was absent in the arm that avoided peanut, and collectively, we found strong evidence for a gene environment interaction involving oral peanut protein exposure and HLA in the observed IgG4 response to peanut. We should note that the HLA-DQA1*01:02 allele is not associated with baseline selection criteria of egg allergy and eczema (*P* = 4.82 × 10^–1^ and *P* = 2.76 × 10^–1^, respectively), and the association between psIgG4 and HLA-DQA1*01:02 is independent of these baseline selections (*P* = 9.49 × 10^–5^, adjusted for egg allergy and eczema). Additionally, this association at 60 months is also independent of peanut-sensitization status as baseline (*P* = 3.48 × 10^–2^ in *n* = 66 sensitized and *P* = 2.38 × 10^–4^ in *n* = 201 nonsensitized at baseline).

While prior studies in the general population have identified the HLA locus as perhaps the locus most consistently associated with food allergy, the specific associations appear to be unique by food type. The associations of the HLA genetic variants and PA allergy reported in prior epidemiological studies of PA are summarized in Supplemental Table 11 along with the association results for PA in the avoidance arm and psIgG4 levels in the consumption arm of the LEAP study. Our peak SNP, rs17612852, in the LEAP study shows LD with previously published SNPs (D′ range: 0.61–0.99). With respect to HLA alleles, the most consistent reported associations are between DQA1*01:02 (OR = 1.8–1.9, minimum *P* = 4.5 × 10^–8^) and PA risk (2, 3); this is exactly the same allele identified by this work. Importantly, peanut exposure in those population studies is uncontrolled, and none of the large investigations into the genetics of PA to date has included any measure of exposure to peanut. Sensitization to peanut and subsequent allergy in these studies could largely be attributed to occurring through skin exposure, and this elicits predominantly IgE responses. Notably, we also observed nominal association with PA in the avoidance group (OR = 1.99, *P* = 4.37 × 10^–2^) of LEAP, where any exposure to peanut would be through the skin, given the avoidance of oral consumption. It is therefore remarkable that the same HLA allele documented to be a risk allele for PA in the setting of likely skin environmental exposure is found in our investigations to be associated with increased psIgG4 in the setting of controlled oral administration.

Since the primary known function of the HLA class II molecules is to bind specific antigenic peptides and present them for immune recognition, these data suggest that an interaction between mode of exposure and a permissive HLA gene may be responsible for determining the clinical outcome of PA. A key implication of these results is that individuals carrying the HLA-DQA1*01:02 allele may preferentially present particular peanut-allergenic peptides for immune recognition, so although those individuals are at increased risk for allergy, they may also be at increased likelihood of therapeutic benefit from oral exposure. This interpretation is bolstered by our finding that the relationship between high IgG4 levels, oral peanut consumption, and the HLA-DQA1*01:02 allele is predominantly attributable to a single allergenic peanut protein, Ara h 2, which is the most important of the 16 peanut allergens accepted by the World Health Organization, with the vast majority of peanut-sensitive patients recognizing this allergen (11). A pattern of response targeted to Ara h 2 is reflected in the IgG4 antibody specificity where the only epitopes identified at an FDR of less than 0.05 were for Ara h 2.

The HLA region contains a high degree of LD across multiple loci, presenting challenges to interpretation of individual associations such as our findings with a specific allele at the DQA1 locus. Given that DQA1*01:02 frequently coexists on a single haplotype with DQB1*06:02 together with specific DRB1 alleles, we sought to extend the association tests focusing on haplotypes across these genes. Unfortunately, alleles at DRB1 failed our quality control (QC) threshold (83.86% imputation quality), but it was included within the haplotype framework to help dissect the role of a known ancestral haplotype versus a single HLA allele in PA risk (Table 2 and Supplemental Table 4). We found that the strongest effect for psIgG4 was for the DQA1*01:02~DQB1*06:02~DRB1*15:01 haplotype (*P* = 2.94 × 10^–3^); additionally, the DRB1*15:01 allele was itself associated with psIgG4 (*P* = 2.03 × 10^–3^). Therefore, it is not possible to attribute the specific Ara h 2 immune response related to the DQA1*01:02 allele to a single HLA molecule (e.g., HLA-DQ), since other linked class II molecules on this haplotype — or indeed, combinations of alleles — are coexpressed. Intriguingly, we found that a closely linked SNP variant in the HLA-DQ region was also highly associated with IgG4 levels in the LEAP peanut consumption cohort. The major contribution of this variant appears to influence levels of expression of *HLA-DQB1* and *HLA-DQA1* transcripts, both in lymphocytes and in gut tissue specimens, although this does not preclude the presence of other eQTLs in the HLA region. Thus, it is possible that the genetic contribution of HLA in the elevated IgG4 response in the LEAP consumers involves both increased expression and a specific allelic genotype, leading to preferential presentation of Ara h 2 epitopes for immune recognition.

The highly specific relationship between Ara h 2 IgG4 and HLA DQA1*01:02 is in marked contrast to our findings with another allergy-related gene, *MALT1*, found to be associated with progression to PA in the LEAP avoiders. In our prior work, we showed that the *MALT1* association with PA allergy risk is further reflected in higher total psIgE levels and the number of major allergenic components (Ara h 1, Ara h 2, and Ara h 3). Here, we show that these associations are nonspecific and strong across each individual component titer as well. These data highlight 2 distinct modes of genetic contributions to the determination of peanut immune responses: an antigen-specific element (exemplified by HLA) that is exposure dependent and a broader property of permissive allergic immunity that is represented by *MALT1*.

This study has several limitations. As noted above, LD within the HLA region limits our ability to attribute the presentation of peanut epitopes to individual HLA molecules on the haplotype represented by HLA-DQA1*01:02, and the relationship between this particular allelic genotype and the associated SNP variant that is also associated with IgG4 is not entirely clear. We are unable to demonstrate that the HLA-DQA1*01:02 allele is directly associated with tolerance or whether there is a dose response to level of consumption. This limitation in studying the effect of HLA status on tolerance and a dose response is due to the almost complete protection against PA conferred by early consumption and the widespread consumption of high levels of peanut, respectively. It would be important to examine the effect of HLA status on protection against PA in studies where lower amounts of peanuts were consumed than in LEAP and where more children developed PA. Although our individual time point analysis shows nonsignificant associations between HLA-DQA1*01:02 and IgG4 to Ara h 2 at 72 months, we were unable to ascertain the specific reason behind it. One possible explanation behind this effect could be cessation of peanut consumption at 60 months. The HLA alleles used in our analyses were imputed using aligned sequence reads from WGS data using state-of-the-art and often implemented approaches (12); to reduce false inferences, we limited our analyses using stringent imputation quality metrics. Validation in the future using HLA sequencing approaches may be useful. Finally, we recognize that generalizability may be limited because all of the LEAP participants were at high risk for PA in contrast to those in general population studies, and since HLA haplotypes are disproportionately distributed among different ethnic groups, the impact across populations needs to be evaluated further.

Despite these limitations, the ability to test for genetic determinants of PA and psIgG4 while explicitly modeling oral exposure to peanut protein in early life has enabled us to identify strong interactions between individual genotype and early peanut consumption in LEAP. Evidence for gene interactions with oral exposure in psIgG4 levels is noted as early as 12 months and increases through to 30 and 60 months of age after continuous oral peanut protein exposure since infancy. In the absence of early oral exposure, HLA predisposes to PA. Our analysis also supports that these HLA genetic variants primarily drive the IgG4 subtype and are not mediated through HLA associations acting directly on circulating cytokine levels of IL-10, although we cannot rule out an association with IL-10 within the tissue microenvironment. We speculate that levels of psIgG4 in LEAP consumers may have potentially more to do with other factors, including but not limited to gut permeability and antigen presentation, that could lead to permissive immune recognition of particular peanut peptides, which is fundamental to driving differential immune responses, depending on the mode and level of environmental exposure.

## Methods

### Study participants and phenotypes.

The LEAP genetics study data set includes participants from the LEAP randomized controlled trial (6) who consented to genetics studies and completed the trial according to the assigned group of dietary peanut consumption or avoidance. The sample set consisted of *n* = 542 per protocol participants stratified into peanut consumption (*n* = 267) and peanut avoidance (*n* = 275) groups. Numerous immunologic markers,such as skin-prick testing and measurements of peanut-specific IgE, IgG, and IgG4 levels in addition to PA were assessed among the LEAP participants. In this study, we have focused on 3 primary phenotypes of LEAP participants, including PA, psIgE, and psIgG4. Among the LEAP participants, PA was ascertained with a positive oral food challenge (OFC) to peanuts at 60 months of age, and immunoglobulin levels were measured in serum by ImmunoCAP assays (Thermo Fisher Scientific) at baseline (>4–11 months) and 12, 30, 60, and 72 months of age (6).

In addition to psIgE and psIgG4, IgE and IgG4 responses to multiple peanut allergenic protein components (i.e., Ara h 1, h 2, h 3, h 8, and h 9) were also measured at all time points through ImmunoCAP assays (6). The lower detection limit was 70 μg/L for psIgG4, 70 μg/L for psIgG, and 0.01 kU/L for both psIgE and component-specific IgEs. In the case of IgG4 to components, the lower limit was less than 70 μg/L, and in this case, we censored the lower limit to be 70/2 or 35 μg/L. IL-10 and IL-4 cytokine levels were measured in plasma samples of consumption group participants at 60 months using the high-sensitivity multiplex platform from Quanterix (https://www.quanterix.com). The lower limit of quantification (LLOQ) for IL-10 was 0.0976 pg/mL and for IL-4 was 0.1952 pg/mL. The values below LLOQ for IL-4 were censored to be 0.1952/2 or 0.0976 pg/mL.

### Study participant characteristics.

Baseline assessment criteria and clinical characteristics of 542 per protocol LEAP participants (8) are described in Table 1. Participants from both peanut-consumption and -avoidance groups were similar in age, sex, and ethnicity. There was an equal proportion of egg-allergic participants, and eczema severity was similar in both peanut-avoidance and -consumption groups. At 60 months, the levels of psIgG4 were higher in the participants of the peanut-consumption group as compared with the participants of the peanut-avoidance group (*P* = < 2.2 × 10^–16^). Levels of IgG4 to individual components Ara h 1, h 2, and h 3 were also strongly different between the 2 groups; no differences were noted for IgG4 to Ara h 8 and 9. While IgE to Ara h 1 and h 2 were higher in the peanut-avoidance group, no differences were noted for psIgE and IgE to Ara h 3, h 8, or h 9. Finally, there was a higher proportion of peanut-allergic participants in the avoidance group compared with consumption group (17.5% vs. 0.4%); there was only a single peanut-allergic participant in the consumption group.

### Genotyping and imputation of HLA variants and quality control.

A detailed account of the sample and variant-based QC methods and the deconvolution of genetic ancestry with self-reported race/ethnicity of LEAP participants was published previously (8). Briefly, WGS and GWAS array data (Illumina InfiniumOmni2-5-8v1-3 array) were available on *n* = 542 LEAP study participants (8). Here, we assessed genetic associations in the HLA region relying on genotyped SNPs, imputed alleles, and haplotypes.

HLA alleles were imputed using HISAT-genotype software that utilizes the hierarchical indexing for spliced alignment of transcripts 2 (HISAT2) alignment system to align DNA sequences using a graph Ferragina Manzini index (12). Aligned reads from the 30× WGS performed using the Illumina HiSeq X System for the HLA region (chr6:28,000,000–35,000,000) were extracted using samtools (version 0.1.19) to prepare the input files for HLA alleles imputation. We were able to impute 339 alleles from 24 HLA class I and class II genes. We filtered out genes with a call rate of less than 95% and limited analysis to those alleles with a frequency of 5% or more for each passing gene, resulting in 38 alleles across 8 genes.

Haplotypes were generated across *HLA-DQA1* and *HLA-DQB1* genes using the Bridging Immunogenomic Data-Analysis Workflow Gaps (BIGDAWG) R package (13) and the imputed HLA alleles from HISAT, above. Only samples with complete haplotype data (i.e., those with alleles at both genes) were retained for analysis, and analysis was limited to those haplotypes with a frequency of 5% or more. Using BIGDAWG, we were able to impute 39 haplotypes across the *HLA-DQA1* and *HLA-DQB1*, of which 7 had a frequency of 5% or more.

In addition to the HLA alleles and haplotypes imputed from the WGS data, we also tested for genetic association with the SNPs in the HLA region. The assembly and variant calling on WGS data were performed using the Starling Small Variant Caller (formerly called the Isaac Variant Caller) (14). Given that the Starling Small Variant Caller has limitations in calling the variants in the hypervariable MHC region accurately, we used genotyped data from the Infinium Omni 2.5 array (version 1.3) that was also generated on all the samples along with WGS data. QC filters, including overall minor allele frequency of 2% or more, overall variant missingness of less than 5%, and a Hardy-Weinberg equilibrium *P* value of 1 × 10^–6^ or more, among all participants were applied to genotyped SNPs. A total of 9742 SNPs across the HLA region passed these QC filters.

### Genetic association analyses at HLA alleles, HLA haplotypes, and SNPs.

The log-transformed (log_10_) values of quantitative traits were used in the association models. At 60 months of age, 48 participants from the avoidance group and 1 participant from the consumption group were ascertained to be peanut allergic. Therefore, associations for PA were assessed in participants from the avoidance group only. The associations for psIgE, psIgG4, and IgE and IgG4 to peanut components (Ara h 1, h 2, h 3, h 8, and h 9) levels were assessed in both avoidance and consumption groups. Discovery analyses were all performed at 60 months, and follow-up analyses were extended to all time points (baseline, 12, 30, and 72 months) using the same models described below. The numbers of the participants included in each model at each time point are shown in Supplemental Table 6.

HISAT-genotype is specifically designed to compute the HLA type of a human using standard WGS data, but does not facilitate tests for association including covariates. Therefore, we called the HLA alleles using HISAT-genotype, but then performed the association testing between the HLA alleles and phenotypes under a dominant model (i.e., carrier vs. noncarrier for each allele) in PLINK 1.9 (15) using the first 5 principal components (PCs) as previously documented (8), age in months at entrance, and sex as covariates. A logistic model was used for PA, and linear models were used for all the quantitative traits. With respect to the haplotypes, while BIGDAWG performs case-control analyses, it does not allow for quantitative trait analyses. Additionally, BIGDAWG limits the inclusion of covariates in the association testing (13). Therefore, we generated haplotypes using BIGDAWG, but tested them in PLINK 1.9 under a dominant model (i.e., carrier vs. noncarrier for each haplotype) as described above for the alleles.

For SNPs mapping to the HLA region (chr6:28,477,797-33,448,354/GRCh37), tests for association were performed in PLINK 1.9 (15), testing each SNP under an additive model. The exact analysis models and covariates for PA, psIgE, and psIgG4 stated above for the allele and haplotypes were used. Regional LocusZoom plots (http://locuszoom.org/) were generated on all QC-passing variants. We used the threshold of *P* < 5.13 × 10^–6^ to correct for the multiple testing (i.e., 0.05/9742 SNPs).

### Gene-expression analyses.

We queried whether associated SNP(s) were also identified as cis-eQTLs in GTEx (https://www.gtexportal.org/home/datasets) and eQTLGen (https://www.eqtlgen.org/) databases. For GTEx (version 7, dbGap accession phs000424.v7.p2), significant variant gene association results were downloaded from the GTEx portal for esophagus (gastrophageal junction, mucosa, and muscularis), stomach, small intestine, colon (sigmoid and transverse), and whole blood tissues. Normalized effect sizes (NES) of the minor allele were used to interpret the direction of effect on gene regulation and aligned to the same allele used in our analysis to facilitate comparison. Additionally, significant cis-eQTL results for whole blood were downloaded from eQTLGen, and *z* score values with respect to minor allele were used to interpret the direction of effect on gene regulation.

### RNA-Seq and multivariate models.

CD4^+^ T cells were isolated by negative selection from V60 banked PBMCs from LEAP subjects from the avoidance (*n* = 63) and consumption arms (*n* = 23). Cells were cultured for 6 hours in RPM1 media with 10% commercial human serum and RNA isolated using the RNeasy Plus Mini Kit (QIAGEN). Colon biopsies were collected from uninflamed tissue during standard-of-care colonoscopy from 28 healthy controls or patients with Crohn’s disease or ulcerative colitis.

Biopsies were stored in RNAlater (Thermo Fisher), and total RNA was isolated by homogenizing tissue in QIAzol lysis reagent (QIAGEN) followed by column purification using the miRNeasy kit (QIAGEN). Sequencing libraries were constructed from total RNA using the SMART-Seq, version 4, Ultra Low Input RNA Kit for Sequencing (Takara) and the Nextera XT DNA Sample Preparation Kit (Illumina) to generate Illumina-compatible barcoded libraries. Dual-index, single-read sequencing of pooled libraries was carried out on a HiSeq2500 sequencer (Illumina) with 58 base reads at a target depth of 5 million reads per sample. Base calls were processed to FASTQs, and processed reads were aligned using the TopHat aligner (version 1.4.1; ref. 16) with the GRCh38 reference genome and gene annotations from ensemble release 77. Gene counts were generated using HTSeq-count (version 0.4.1; ref. 17) and quality metrics applied.

A total of 86 libraries from CD4^+^ T cells and 28 colon biopsy libraries passed QC. Normalized read counts from the *DQB1*, *DQA1*, *DRB1*, *DRA*, *DPA1*, and *DPB1* genes were stratified by genotype of DQA1*01:02 carrier status, and associations in T cell data were analyzed by linear regression using PLINK 1.9, using an additive model for SNP genotype association and a dominant model for association with DQA1*01:02 carrier status. Sex, race, allergy status, treatment arm, and cell viability were included as covariates. Association of read counts from colon RNA-Seq data was calculated using a Mann-Whitney *U* test. Multivariate linear regression modeling was used to assess the contribution of SNP genotype and DQA1*01:02 carrier status to DQB1 expression. The log_10_ HLA-DQB1 measurements were regressed with the SNP genotype, DQA1*01:02, treatment group, and separately with the disease group. Mutually adjusted type 3 *P* values were reported for each term in the models (Figure 2).

### Fine-mapping and colocalization.

We used the Bayesian regression method sum of single effects (18) (SuSiE, susieR R package version 0.9.0) to fine map the HLA class II locus for traits (psIgG4 and Ara h 2) and eQTLs (*HLA-DQA1* and *HLA-DQB1*). SuSiE reports credible sets, sets of variants that include at least 1 causal variant with high probability at specified thresholds of coverage. For each analysis, linear model results from the PLINK association analysis were combined with the LD matrix based on the exact set of included individuals (e.g., all consumers for the traits and 86 samples with RNA data for the eQTLS) in SuSie under a coverage threshold of 0.95. The genetic variants were limited to 1908 SNPs and 14 HLA alleles mapping to the class II region.

As an additional step, direct pairwise colocalization analysis between the trait and gene expression was performed using the R package coloc (19). The method uses approximate Bayes factor computations and tests pairwise colocalization of variants in GWAS data sets with eQTLs. It generates 5 posterior probabilities (PPs), namely, PP0, PP1, PP2, PP3, and PP4, for the locus by using the evidence for competing hypotheses of either no colocalization or colocalization (19). A PP3 value of approximately 75% indicates evidence against colocalization. In contrast, a PP4 value of approximately 75% supports evidence of colocalization. The test also examines the PP4 for each SNP within the region for the likely causal variant. Coloc evaluated the trait (psIgG4) against both *HLA-DQA1* and *HLA-DQB1* eQTL associations for 1908 SNPs and 14 HLA alleles mapping to the class II region.

### Temporal patterns of association for HLA-DQA1*01:02 and MALT1.

The unadjusted, stratified mean levels of psIgG4 and IgG4 and psIgE and IgE to peanut allergenic components at baseline (>4–11 months) and 12, 30, and 60 months were compared within the carriers and noncarriers of HLA-DQA1*01:02 and *MALT1* rs57265082 in all the available data. A mixed effects linear model of each outcome shown in Figure 3 was fit separately with the following predictors: treatment group, age at assessment, HLA-DQA1*01:02 carrier status, the interaction effect between the HLA-DQA1*01:02 carrier status and treatment group, and the interaction effect between age at assessment and treatment group. An AR1 covariance structure was assumed to account for the repeated measurements over the visits. Unadjusted mean values with bootstrapped 95% CIs are displayed in Figure 3. Similar models were performed for the levels of psIgE and IgE responses to peanut components at all time points for *MALT1* carriers and noncarriers among peanut avoiders and consumers.

### Sequential linear epitope assay.

For a subset of 323 children (Supplemental Table 9) in the LEAP trial, the bead-based epitope assay (BBEA) was used to measure IgE and IgG4 antibodies binding to 64 sequential (linear) epitopes in Ara h 1 (*n* = 34), Ara h 2 (*n* = 16), and Ara h 3 (*n* = 14) proteins, as previously described (20). The experiment was run in triplicate with subsequent QC, and epitope binding was qualified as described (20).

### Longitudinal analysis using mixed effect models.

Changes in ses-IgE and ses-IgG4 profiles during the 5 years of the LEAP trial were compared between allele carriers and noncarriers under the dominant (binary) allele association model. Binding to 64 epitopes was modeled using linear mixed effect models, including variant, PA outcome at 60 months (not allergic, sensitized, allergic) and at other visits as well as its 3-way interaction as fixed factors and a random intercept for each participant. The first 5 genetic PCs were also included as time-invariant covariates in both epitopes and *z* score models. Overall binding to linear epitopes specific to each Ara h 1, h 2, and h 3 were generated by combining the binding to each epitope of that protein in a *z* score: ses-Ara h 1, ses-Ara h 2, and ses-Ara h 3 (21).

The epitope data were modeled in the limma (22, 23) framework, appropriate for high-throughput data. The *z* scores were modeled using the nlme package (24). For each group, we estimated the marginal mean and 95% CI and used contrasts to estimate the significance of (a) the epitope expansion defined as the change in binding from baseline to each visit and (b) differences between epitope expansion of allele carrier and noncarrier at given time points. *P* values of the hypothesis tested were adjusted for multiple comparisons (across epitopes) using the Benjamini-Hochberg procedure that controls the FDR.

### CCA.

Besides the univariate model for each epitope, we used a multivariate CCA, also known as canonical variates analysis (25). CCA is a method for exploring the relationships between 2 multivariate sets of variables, capturing variability of correlated data, and avoiding multiple testing correction. We applied it to determine whether the multivariate (many epitopes) distribution of the epitope binding was associated with the genotype under the dominant (binary) association model. This was carried out using all 64 epitopes and with 3 independent models that evaluated the effect of the alleles on the binding of epitopes in the Ara h 1, Ara h 2, and Ara h 3 proteins at each time point. To correct for population structure, the binding was adjusted for 5 PCs through a linear model prior to analysis. In the case of a single explanatory variable (allele), CCA determines a linear combination of the epitope (the canonical variate) that maximizes the explained variance of the multivariate distribution and the correlation with the vector of predictors (HLA-DQA1*01:02 or rs57265082). The association of the set of epitopes with the allele is then tested using the Wilks’ lambda test (26) and permutation test (*n* = 10,000 permutations; computations were performed with R package CCP; https://CRAN.R-project.org/package=CCP). Significance indicates that the variant is globally associated with the binding of the epitopes. There was complete agreement between the significance calls (*P* < 0.05) of the Wilks’ lambda and the permutation *P* values.

### Data and materials availability.

WGS and GWAS data are available through the NCBI’s dbGAP database (phs001851.v1.p1). Other data sets are available through TrialShare, a public website managed by the Immune Tolerance Network (https://www.itntrialshare.org).

### Study approval.

The trial was approved by an IRB (National Research Ethics Service Committee London–Fulham) and was overseen by the Allergy and Asthma Data and Safety Monitoring Board of the National Institute of Allergy and Infectious Diseases. All participants included in the RNA-Seq analysis provided informed consent, and the study was approved by the Benaroya Research Institute IRB (IRB 10090). The Research Ethics Committee reference number for the original LEAP Study is 04/Q0403/13.

## Author contributions

KK, SG, HTB, IR, and GS performed analyses. DL, GDT, KCB, HAS, MSF, GL, GTN, KC, and RAM were involved in the design of the study. KK, SG, HTB, MSF, IR, GL, GTN, KC, and RAM wrote the manuscript. DL, GDT, KCB, and HAS provided critical feedback and edited the manuscript.

## Supplementary Material

Supplemental data

## Figures and Tables

**Table 2:**
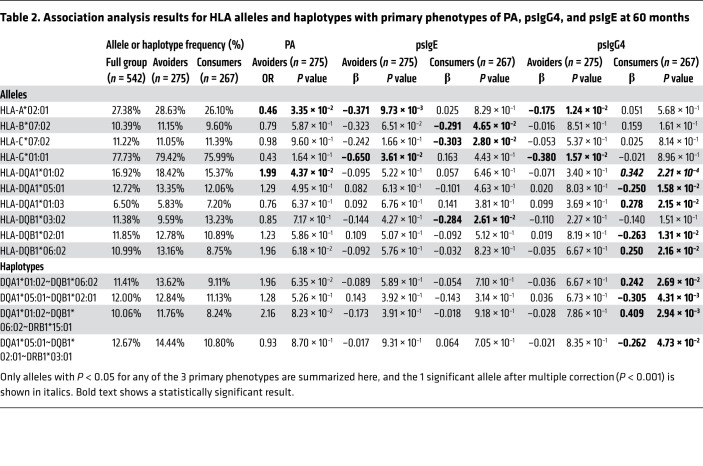
Association analysis results for HLA alleles and haplotypes with primary phenotypes of PA, psIgG4, and psIgE at 60 months

**Table 1:**
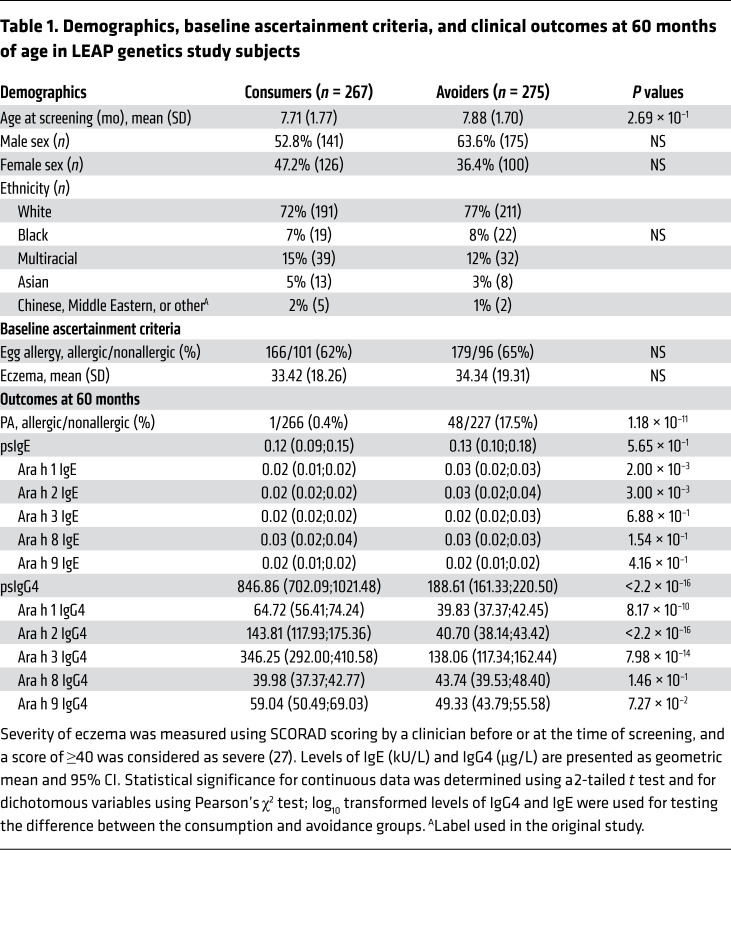
Demographics, baseline ascertainment criteria, and clinical outcomes at 60 months of age in LEAP genetics study subjects

## Version 1 01/04/2022

Electronic publication
