# Supplementary material for: HLA alleles and sustained peanut consumption promote IgG4 responses in subjects protected from peanut allergy
Source: J Clin Invest. 2022 Jan 4;132(1):e152070. doi: 10.1172/JCI152070 (PMC8718139; doi:10.1172/JCI152070)
Supplement: Supplemental data [file jci-132-152070-s070.pdf]

Supplemental figures and figure legends

Supplementary Figure 1: Regional association plots of HLA SNP associations with primary phenotypes of peanut specific IgG4 (psIgG4), peanut specific IgE (psIgE) and PA in LEAP consumption and avoidance group at 60 months. Red line shows statistically significant association (P=5.13x10-6, after correcting for 9,742 SNPs in the HLA region). Purple diamonds are the SNPs with lowest P-value for each trait.

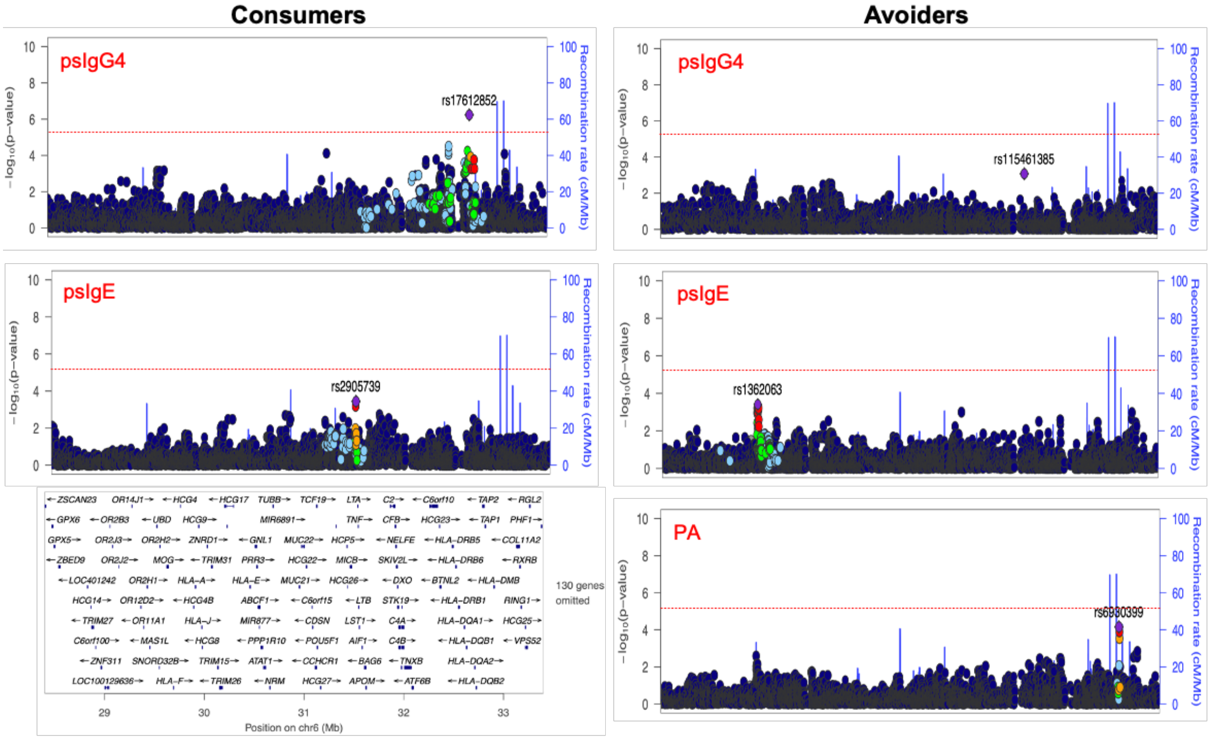

Supplementary Figure 2: Regional association plots of HLA SNP associations with psIgG4 showing **[A]** the primary analysis and **[B]** a conditional analysis where SNPs are tested conditioned on the primary variant rs17612852.

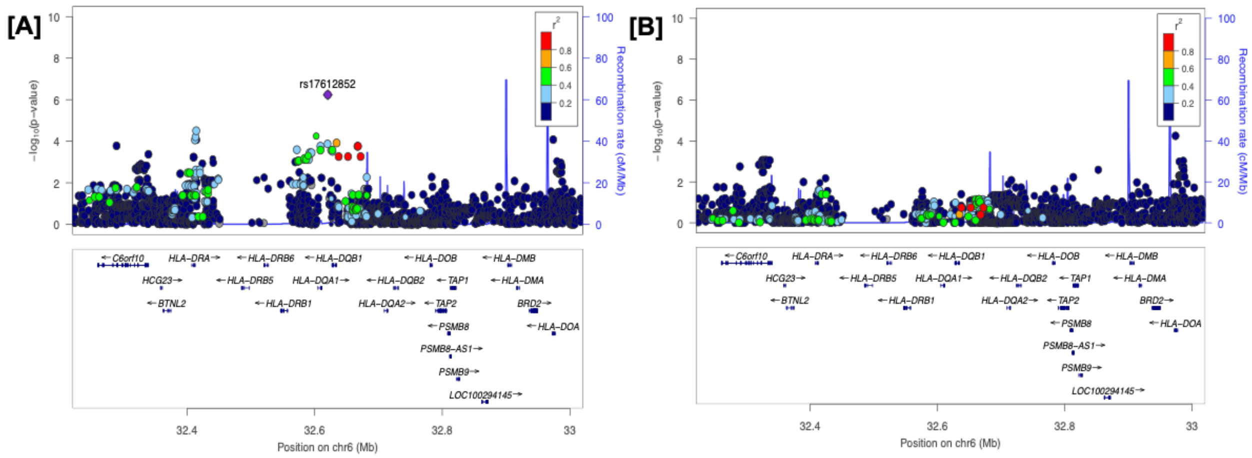

Supplementary Figure 3: Regional association plots the HLA Class II region. Top row is the association for log10(psIgG4) and log10(Ara h 2) at 60 months. Rows 2-4 are the eQTLs for the same variants for each of 7 genes expressed in the CD4 T cells. The color of each dot represents its correlation to the peak variant rs17612852 (see r2 legend). The purple diamond represents index variant rs17612852, and the blue box is drawn around HLA-DQA1*01:02. Red dashed line represents Bonferroni thresholds for the trait associations ($p<5.13x10^{-6}$) and eQTLs ($p<3.71x10^{-6}$).

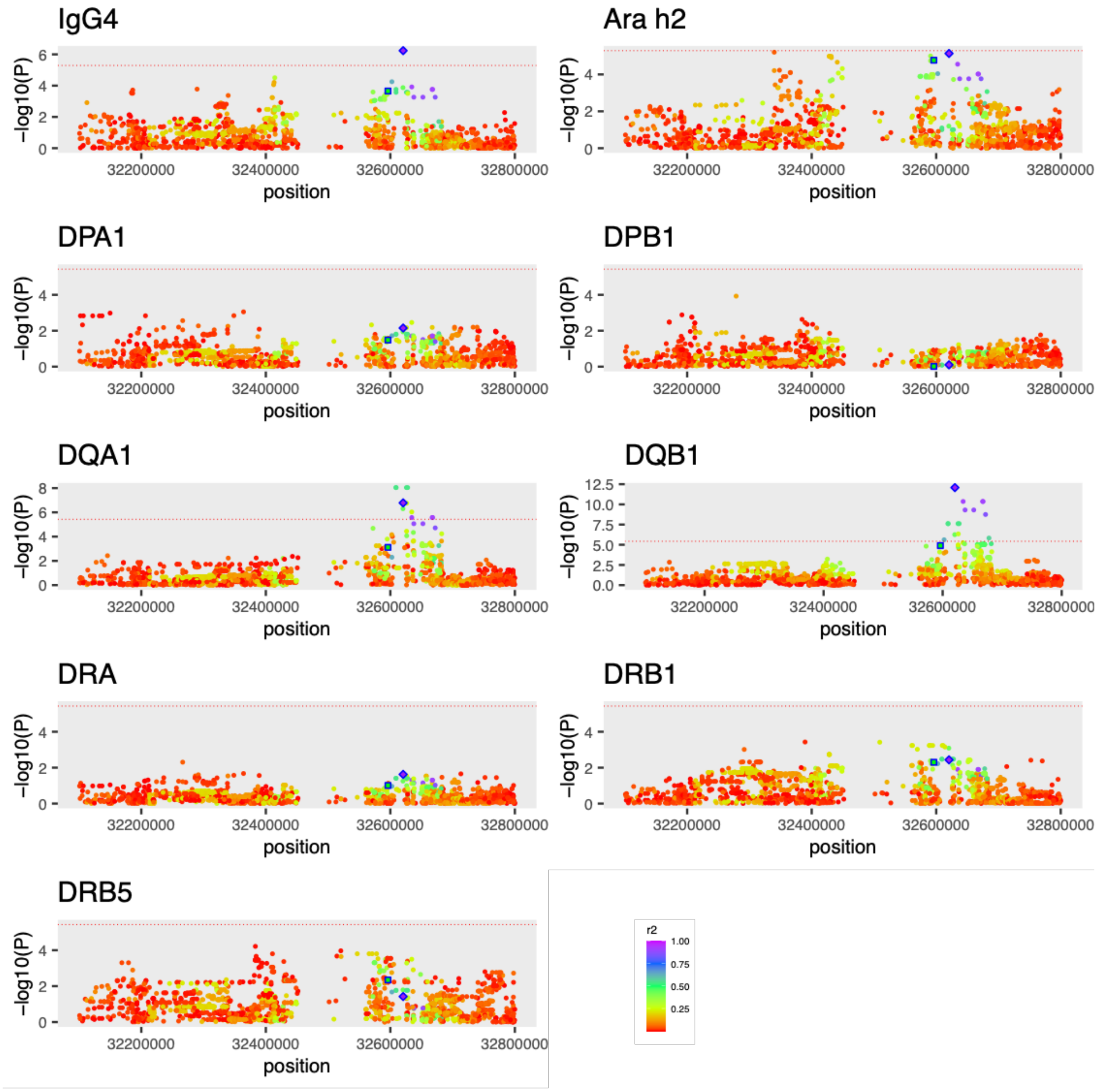

Supplementary Figure 4: Co-localization analysis between the eQTLs for Class II gene expression (*HLA-DQA1* and *HLA-DQB1*) in CD4 T cells and phenotype associations noted for psIgG4. **Panel A** shows the correlation between the p-values noted for the IgG4 associations and eQTLs for each variant for DQA1 (*upper*) and DQB1 (*lower*); the blue diamond is sentinel variant rs17612852. The p-value for the IgG4 association is same in the upper and lower plot, but the eQTL p-values differ. It is noted that for DQA1 (*upper*), the strongest IgG4 SNP, is not a perfect match for the strongest DQA1 gene eQTL; there are several SNPs with stronger regulatory evidence for gene expression (green oval). In contrast for DQB1, the strongest eQTL SNP perfectly matches the strongest IgG4 SNP rs17612852. **Panel B** is the SuSie fine-mapping for *HLA-DQB1* eQTLs showing a single credible set (CS1) with the sentinel variant rs17612852 identified as the target variant. This is additional evidence that there is perfect co-localization between the IgG4 association and regulatory evidence for HLA-DQB1. Also marked is HLA-DQA1*01:02 which is not identified in a credible set for *HLA-DQB1* expression.

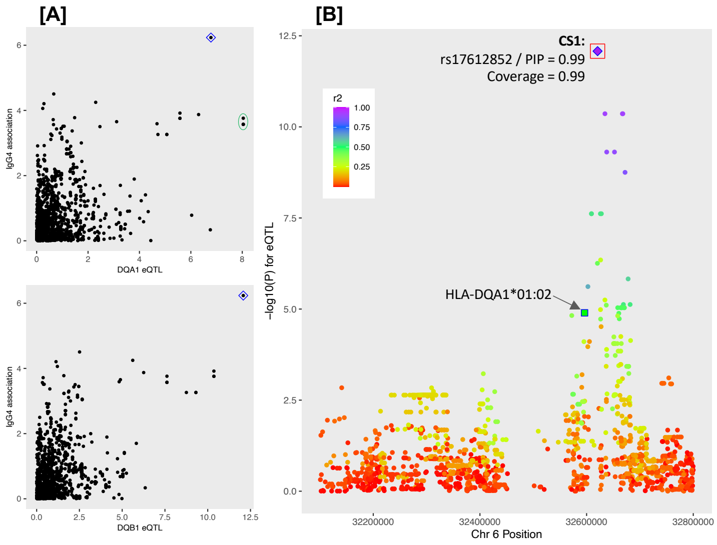

Supplementary Figure 5: Associations of HLA-DQA1*01:02 and *MALT1* (rs57265082) with psIgG4 and psIgE over time. Figures show the -log(P-value) across the visits, and tables have the effect sizes for the variants for log10(psIgE) and log10(psIgG4). The effect sizes represent the difference in the mean between the two groups (carrier vs. non-carrier) on the log10 scale.

[A] HLA-DQA1*01:02 / LEAP Consumption Group

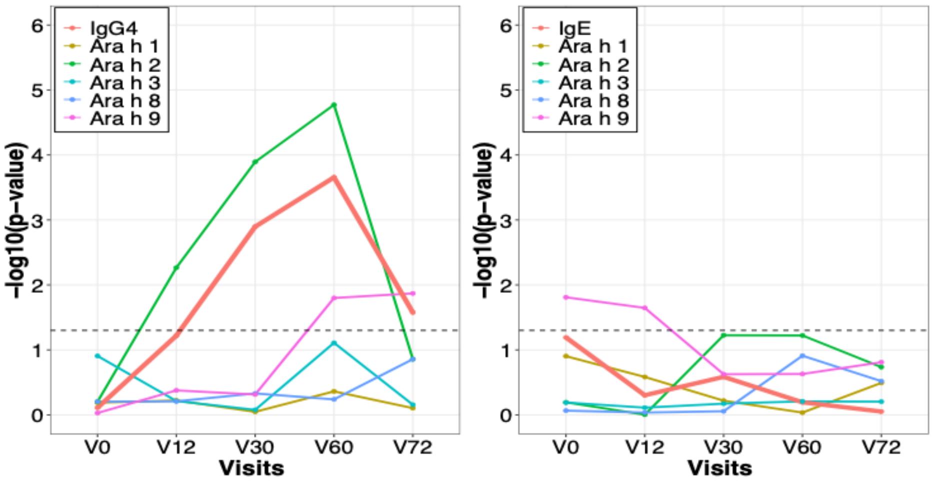

Effect Sizes

Pheno	V0	V12	V30	V60	V72
IgG4	0.006	0.147	0.325	0.342	0.213
Ara h 1	0.006	-0.026	-0.011	0.053	-0.014
Ara h 2	-0.002	0.207	0.436	0.420	0.124
Ara h 3	0.068	-0.036	-0.020	0.148	0.035
Ara h 8	-0.002	-0.003	-0.023	-0.019	0.069
Ara h 9	0.000	0.021	0.049	0.188	0.250

Pheno	V0	V12	V30	V60	V72
IgE	-0.244	-0.085	0.140	0.058	-0.020
Ara h 1	-0.139	-0.118	-0.044	-0.006	-0.069
Ara h 2	0.028	0.001	0.160	0.124	0.097
Ara h 3	-0.037	-0.027	0.039	0.039	-0.046
Ara h 8	-0.006	-0.004	0.010	0.213	0.181
Ara h 9	0.092	0.159	0.084	0.085	0.120

[B] MALT1 (rs57265082) / LEAP Avoidance Group

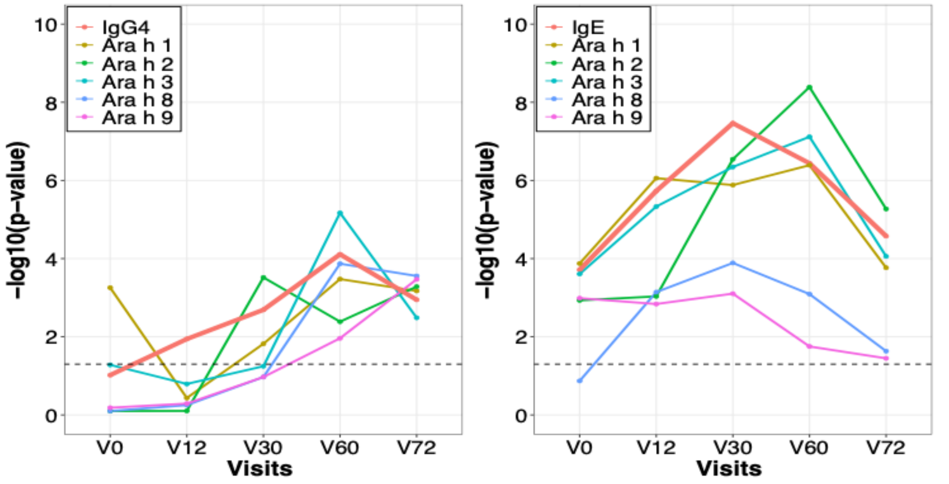

Effect Sizes

Pheno	V0	V12	V30	V60	V72
IgG4	0.032	0.094	0.271	0.429	0.360
Ara h 1	0.077	0.025	0.113	0.160	0.131
Ara h 2	-0.002	-0.003	0.131	0.130	0.163
Ara h 3	0.104	0.092	0.194	0.515	0.368
Ara h 8	-0.001	-0.010	0.073	0.265	0.292
Ara h 9	-0.008	0.022	0.117	0.208	0.303

Pheno	V0	V12	V30	V60	V72
IgE	0.639	0.865	1.101	1.099	0.960
Ara h 1	0.476	0.678	0.761	0.876	0.678
Ara h 2	0.265	0.360	0.796	1.186	0.957
Ara h 3	0.349	0.500	0.581	0.757	0.578
Ara h 8	0.067	0.131	0.243	0.555	0.505
Ara h 9	0.191	0.227	0.304	0.214	0.225

Supplementary Figure 6: Associations of HLA-DQA1*01:02 and *MALT1* (rs57265082) with psIgG4 and psIgE over time in Consumption and Avoidance groups. Figures show the -log(P-value) across the visits, and tables have the effect sizes for the variants for log10(psIgE) and log10(psIgG4). The effect sizes represent the difference in the mean between the two groups (carrier vs. non-carrier) on the log10 scale.

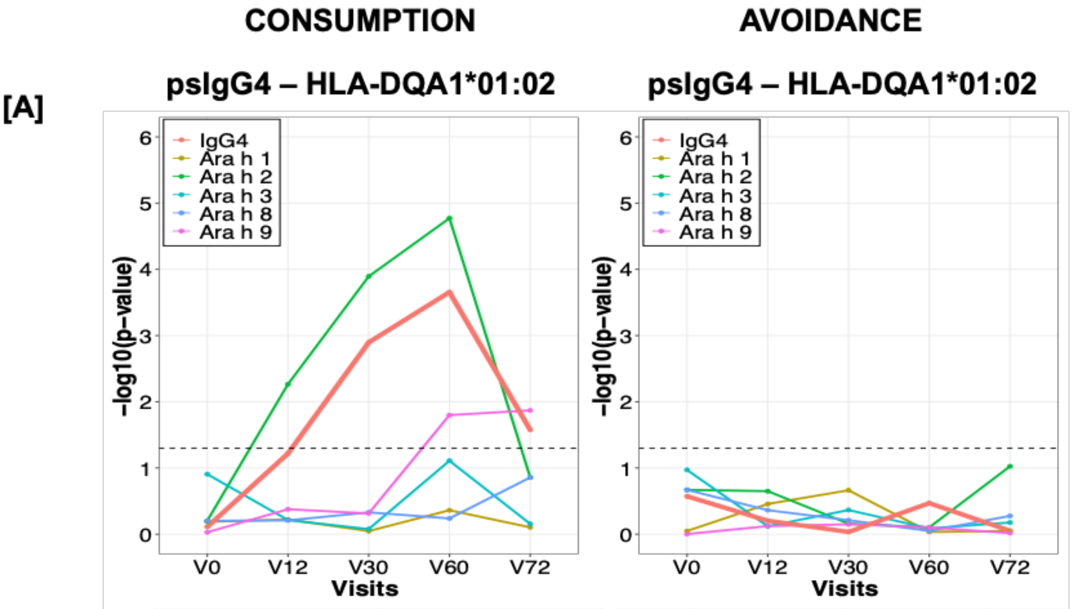

Effect Sizes

Pheno	V0	V12	V30	V60	V72
IgG4	0.006	0.147	0.325	0.342	0.213
Ara h 1	0.006	-0.026	-0.011	0.053	-0.014
Ara h 2	-0.002	0.207	0.436	0.420	0.124
Ara h 3	0.068	-0.036	-0.020	0.148	0.035
Ara h 8	-0.002	-0.003	-0.023	-0.019	0.069
Ara h 9	0.000	0.021	0.049	0.188	0.250

Pheno	V0	V12	V30	V60	V72
IgG4	-0.012	-0.012	-0.006	-0.071	0.011
Ara h 1	-0.002	0.018	0.040	-0.003	-0.004
Ara h 2	0.005	0.011	0.008	0.008	0.048
Ara h 3	0.061	-0.014	0.055	-0.018	-0.038
Ara h 8	0.005	0.010	0.016	0.006	0.035
Ara h 9	0.000	0.008	0.019	-0.015	0.003

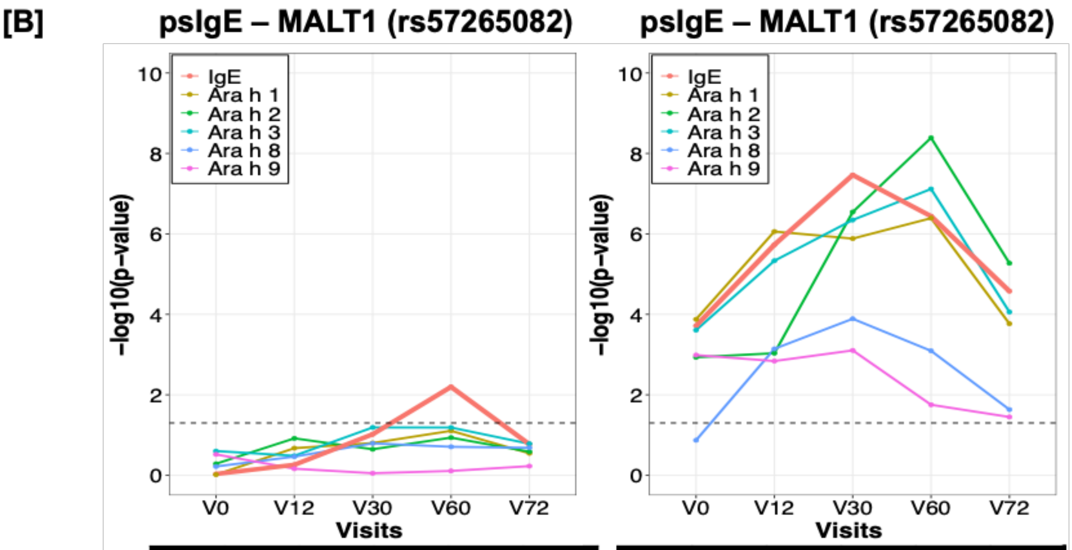

Effect Sizes

Pheno	V0	V12	V30	V60	V72
IgE	-0.015	-0.099	-0.272	-0.443	-0.250
Ara h 1	0.005	-0.170	-0.154	-0.141	-0.091
Ara h 2	-0.051	-0.160	-0.131	-0.132	-0.097
Ara h 3	-0.121	-0.119	-0.224	-0.188	-0.168
Ara h 8	-0.025	-0.042	-0.124	-0.235	-0.290
Ara h 9	0.047	0.033	-0.012	-0.025	-0.054

Pheno	V0	V12	V30	V60	V72
IgE	0.639	0.865	1.101	1.099	0.960
Ara h 1	0.476	0.678	0.761	0.876	0.678
Ara h 2	0.265	0.360	0.796	1.186	0.957
Ara h 3	0.349	0.500	0.581	0.757	0.578
Ara h 8	0.067	0.131	0.243	0.555	0.505
Ara h 9	0.191	0.227	0.304	0.214	0.225

Supplementary Figure 7: Relative distribution of psIgG4 and psIgE, and IgG4 and IgE to peanut components by carrier status at HLA-DQA1*01:02 and *MALT1* SNP rs57265082, across all visits. Unadjusted mean values of the phenotypes stratified by variants carrier status at each assessment and by treatment group assignment are shown with bootstrapped 95% confidence intervals and P-values. Dashed line at 60 months of age depicts the end of LEAP trial at which all participants avoided peanut consumption for 12 months.

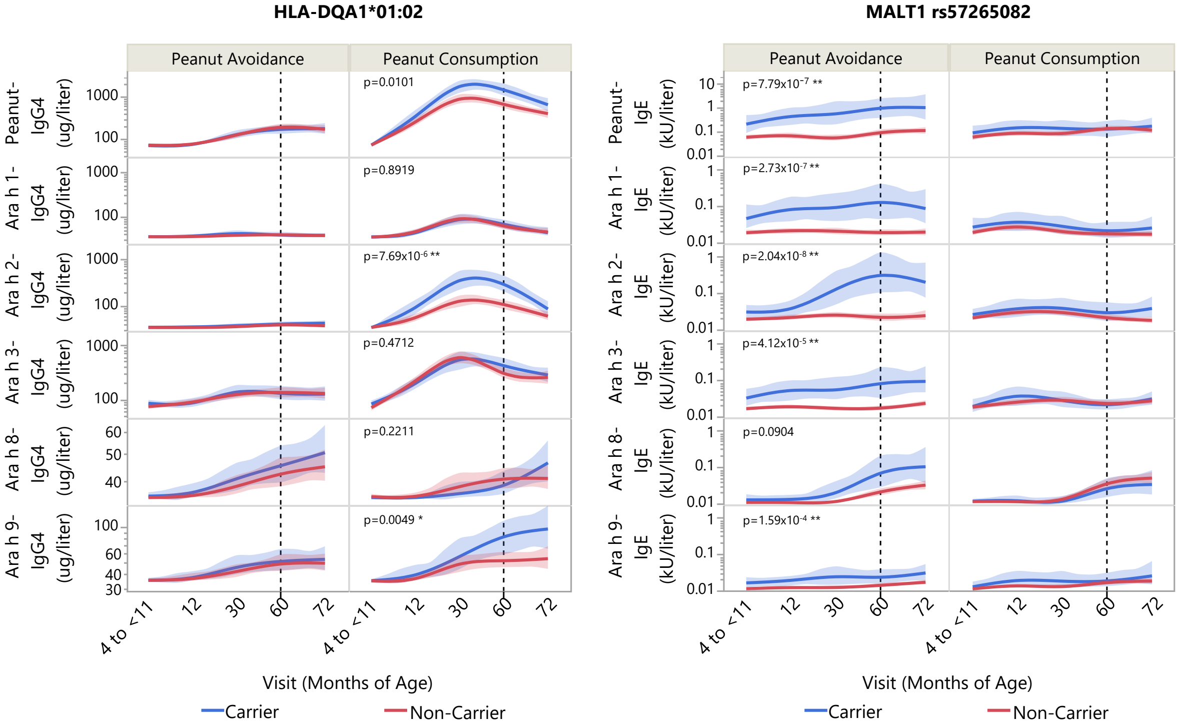

Supplemental Figure 8: Association of mean linear epitope bindings with genotypes at 60 months vs. baseline in peanut exposure groups subdivided by outcome. In **[A and B]** bars represent the changes in expansion of z-scores of mean bindings per peanut component from baseline to visit 60. Stars indicate significance in differences between the two genotypes. **[A]** ses-IgG4 expansion by HLA-DQA1*01:02 genotype. **[B]** ses-IgE expansion by *MALT1* genotype status; error-bars in all panels represent confidence intervals: Mean±95%CI; sample size within each group is represented in brackets.

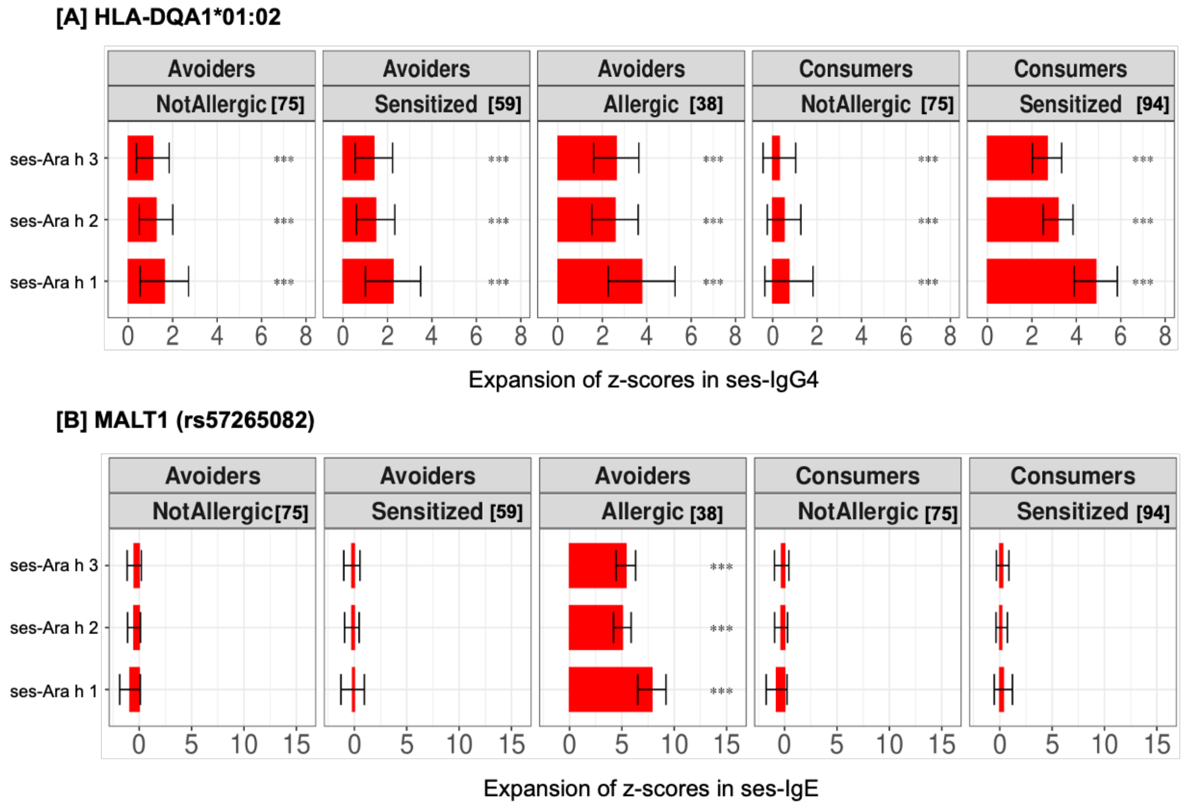

Supplementary Figure 9: Canonical Correlation Analysis (CCA) evaluating the association of the linear epitopes in ses-IgG4 belonging to groups related to Ara h 1, h 2 and h 3 with HLA-DQA1*01:02 and ses-IgE Ara h 1, h 2 and h 3 with *MALT1* rs57265082 over time in the Consumption and Avoidance groups. Dashed lines indicate significance level at p=0.05.

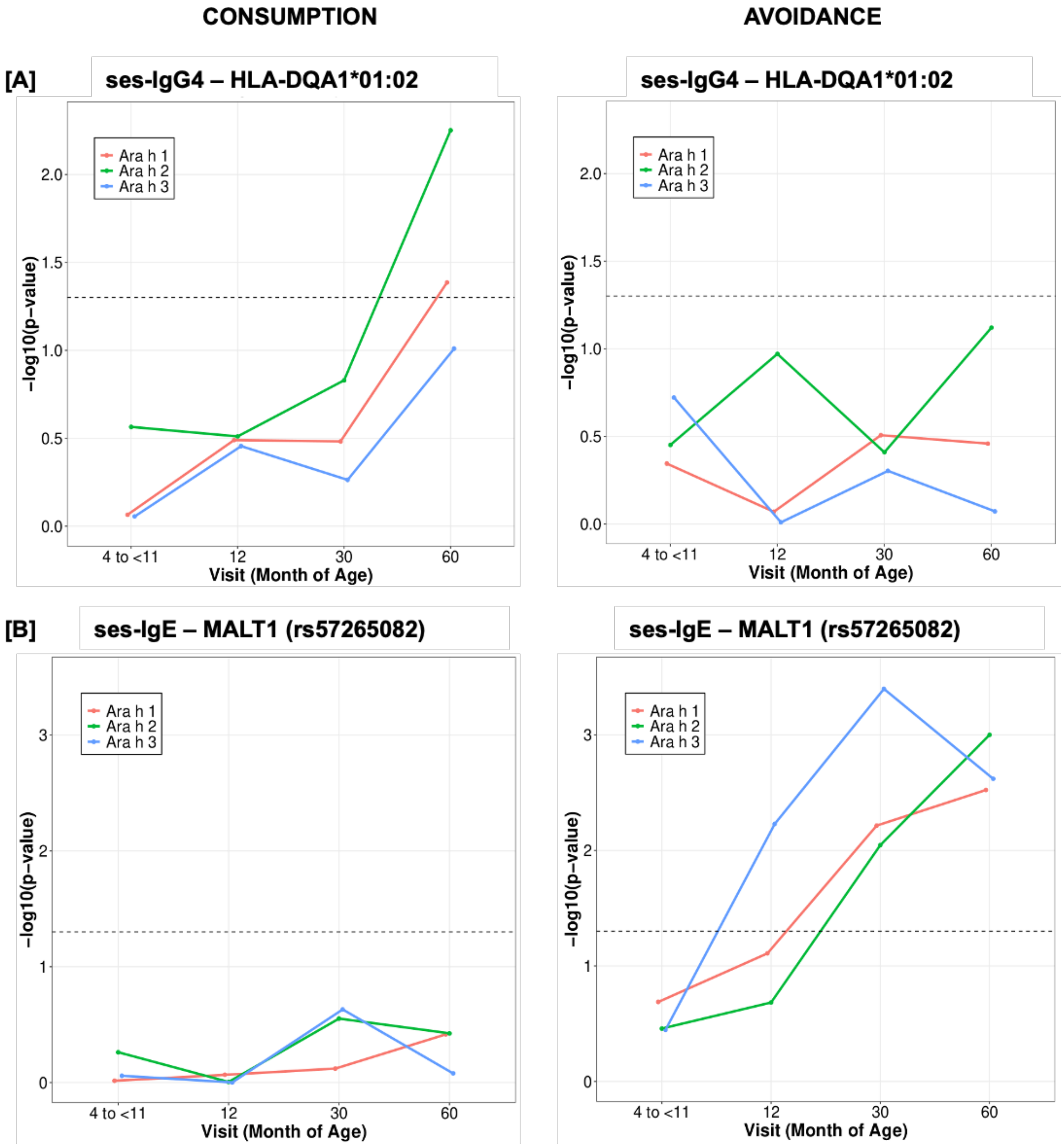

Supplementary Tables:

Table S1: Association analysis results for all 38 HLA alleles with primary phenotypes of peanut allergy, psIgG4 and psIgE at 60 months. Results with $p<0.05$ are bolded.

	**Allele Freq (%)**			**PA**		**psIgE**				**psIgG4**			
	**Full Group**	**Avoiders**	**Consumers**	**Avoiders**		**Avoiders**		**Consumers**		**Avoiders**		**Consumers**	
**Gene*Allele**	**(N=542)**	**(N=275)**	**(N=267)**	**OR**	**P-Value**	**Beta**	**P-Value**	**Beta**	**P-Value**	**Beta**	**P-Value**	**Beta**	**P-Value**
HLA-A*03:01	11.41%	11.76%	11.04%	0.98	9.52E-01	-0.192	2.52E-01	-0.104	4.65E-01	0.018	8.23E-01	-0.061	5.75E-01
HLA-A*01:01	14.48%	15.10%	13.86%	1.62	2.05E-01	0.198	2.21E-01	0.029	8.29E-01	-0.008	9.20E-01	-0.102	3.17E-01
HLA-A*24:02	9.42%	7.45%	11.45%	0.39	1.42E-01	-0.075	7.19E-01	0.060	6.82E-01	-0.042	6.82E-01	0.183	9.63E-02
HLA-A*11:01	5.95%	4.71%	7.23%	1.82	2.69E-01	0.308	2.26E-01	-0.070	6.94E-01	0.243	5.48E-02	0.070	6.09E-01
HLA-A*02:01	27.38%	28.63%	26.10%	**0.46**	**3.35E-02**	**-0.371**	**9.73E-03**	0.025	8.29E-01	**-0.175**	**1.24E-02**	0.051	5.68E-01
HLA-B*07:02	10.39%	11.15%	9.60%	0.79	5.87E-01	-0.323	6.51E-02	**-0.291**	**4.65E-02**	-0.016	8.51E-01	0.159	1.61E-01
HLA-B*08:01	10.20%	11.92%	8.40%	1.72	1.61E-01	0.255	1.40E-01	-0.059	7.14E-01	0.072	4.04E-01	-0.203	1.06E-01
HLA-B*44:02	9.12%	9.04%	9.20%	1.37	4.67E-01	0.163	3.93E-01	-0.184	2.26E-01	-0.066	4.83E-01	-0.064	5.88E-01
HLA-B*44:03	6.27%	6.92%	5.60%	1.04	9.42E-01	0.135	5.16E-01	-0.053	7.64E-01	0.194	6.00E-02	-0.075	5.86E-01
HLA-B*40:01	5.29%	4.81%	5.80%	0.32	1.47E-01	-0.043	8.61E-01	-0.031	8.67E-01	-0.052	6.63E-01	0.024	8.68E-01
HLA-C*07:02	11.22%	11.05%	11.39%	0.98	9.60E-01	-0.242	1.66E-01	**-0.303**	**2.80E-02**	-0.053	5.37E-01	0.025	8.14E-01
HLA-C*07:01	13.83%	15.89%	11.78%	1.54	2.39E-01	0.175	2.74E-01	0.149	2.85E-01	0.061	4.39E-01	0.032	7.58E-01
HLA-C*06:02	8.90%	7.75%	10.04%	2.23	5.68E-02	0.383	6.16E-02	-0.121	4.00E-01	0.029	7.70E-01	-0.164	1.31E-01
HLA-C*04:01	10.64%	11.05%	10.23%	0.59	2.91E-01	-0.101	5.89E-01	-0.113	4.40E-01	0.083	3.63E-01	0.079	4.81E-01
HLA-C*16:01	5.61%	6.01%	5.21%	0.96	9.34E-01	0.068	7.62E-01	0.166	3.86E-01	0.026	8.12E-01	0.048	7.41E-01
HLA-C*03:04	7.45%	7.17%	7.72%	0.39	1.41E-01	-0.176	3.91E-01	0.126	4.36E-01	-0.092	3.59E-01	0.044	7.17E-01
HLA-C*05:01	8.22%	8.91%	7.53%	0.94	8.92E-01	0.020	9.19E-01	-0.105	5.16E-01	-0.068	4.73E-01	0.017	8.89E-01
HLA-C*12:03	5.03%	5.62%	4.44%	0.36	1.90E-01	-0.325	1.78E-01	-0.097	6.48E-01	-0.086	4.68E-01	-0.057	7.21E-01
HLA-G*01:01	77.73%	79.42%	75.99%	0.43	1.64E-01	**-0.650**	**3.61E-02**	0.163	4.43E-01	-0.380	**1.57E-02**	-0.021	8.96E-01
HLA-G*01:04	13.18%	12.88%	13.49%	0.96	9.28E-01	0.009	9.63E-01	-0.073	5.94E-01	0.108	2.44E-01	0.061	5.61E-01
HLA-L*01:01	76.04%	76.95%	75.10%	0.90	8.52E-01	0.101	7.11E-01	-0.249	2.40E-01	-0.128	3.43E-01	-0.203	2.14E-01
HLA-L*01:02	23.96%	23.05%	24.90%	0.64	2.32E-01	0.025	8.72E-01	0.099	3.91E-01	-0.008	9.12E-01	0.132	1.41E-01
HLA-DQA1*03:01	9.18%	7.71%	10.70%	0.94	9.06E-01	-0.166	4.10E-01	-0.231	9.39E-02	-0.170	9.09E-02	-0.067	5.21E-01
HLA-DQA1*05:05	12.43%	9.96%	14.98%	0.90	8.12E-01	0.024	8.98E-01	0.017	8.92E-01	0.067	4.67E-01	0.031	7.50E-01
HLA-DQA1*01:02	16.92%	18.42%	15.37%	**1.99**	**4.37E-02**	-0.095	5.22E-01	0.057	6.46E-01	-0.071	3.40E-01	**0.342**	**2.21E-04**
HLA-DQA1*05:01	12.72%	13.35%	12.06%	1.29	4.95E-01	0.082	6.13E-01	-0.101	4.63E-01	0.020	8.03E-01	**-0.250**	**1.58E-02**
HLA-DQA1*01:01	10.04%	10.15%	9.92%	0.56	2.63E-01	-0.121	5.02E-01	0.010	9.46E-01	-0.022	8.11E-01	-0.034	7.52E-01
HLA-DQA1*01:03	6.50%	5.83%	7.20%	0.76	6.37E-01	0.092	6.76E-01	0.141	3.81E-01	0.099	3.69E-01	**0.278**	**2.15E-02**
HLA-DQA1*02:01	14.24%	15.60%	12.84%	0.92	8.34E-01	0.142	3.60E-01	0.066	6.12E-01	0.069	3.76E-01	-0.127	1.95E-01
HLA-DQA1*03:03	8.60%	8.27%	8.95%	0.94	8.96E-01	0.113	5.53E-01	-0.052	7.33E-01	0.006	9.49E-01	-0.197	8.90E-02
HLA-DQB1*03:01	18.83%	15.79%	21.98%	0.75	4.81E-01	0.106	5.02E-01	0.157	1.67E-01	0.012	8.79E-01	-0.007	9.39E-01
HLA-DQB1*03:02	11.38%	9.59%	13.23%	0.85	7.17E-01	-0.144	4.27E-01	**-0.284**	**2.61E-02**	-0.110	2.27E-01	-0.140	1.51E-01
HLA-DQB1*02:01	11.85%	12.78%	10.89%	1.23	5.86E-01	0.109	5.07E-01	-0.092	5.12E-01	0.019	8.19E-01	**-0.263**	**1.31E-02**
HLA-DQB1*05:01	11.19%	11.09%	11.28%	0.43	9.92E-02	-0.100	5.72E-01	0.109	4.18E-01	-0.003	9.70E-01	0.014	8.88E-01
HLA-DQB1*02:02	11.66%	13.16%	10.12%	0.90	7.99E-01	0.200	2.20E-01	0.198	1.65E-01	0.097	2.36E-01	-0.157	1.48E-01
HLA-DQB1*06:02	10.99%	13.16%	8.75%	1.96	6.18E-02	-0.092	5.76E-01	-0.032	8.23E-01	-0.035	6.67E-01	**0.250**	**2.16E-02**
HLA-DPB2*03:01	65.75%	64.57%	66.94%	0.84	7.21E-01	-0.221	2.57E-01	-0.244	1.22E-01	0.009	9.22E-01	-0.173	1.48E-01
HLA-DPB2*01:01	34.25%	35.43%	33.06%	0.74	4.31E-01	0.067	6.48E-01	-0.079	5.00E-01	0.028	6.96E-01	-0.017	8.51E-01

Table S2: Evaluating the association between psIgG4 and HLA-DQA1*01:01 accounting for PA. Results with p<0.05 are bolded.

	**AVOIDERS**						**CONSUMERS**					
	**ORIGINAL MODEL**		**EXCLUDING SUBJECTS WITH PA**		**PA AS A COVARIATE**		**ORIGINAL MODEL**		**EXCLUDING SUBJECTS WITH PA**		**PA AS A COVARIATE**	
	**N=275**		**N=227**		**N=275**		**N=267**		**N=266**		**N=267**	
**Model components**	**Beta**	**P-Value**	**Beta**	**P-Value**	**Beta**	**P-Value**	**Beta**	**P-Value**	**Beta**	**P-Value**	**Beta**	**P-Value**
Age	0.023	2.57E-01	0.034	1.33E-01	0.025	2.07E-01	0.027	2.54E-01	0.025	2.81E-01	0.025	2.81E-01
Sex	0.061	3.98E-01	0.038	6.24E-01	0.053	4.59E-01	0.212	1.01E-02	0.207	1.20E-02	0.207	1.20E-02
PC1	-1.255	1.29E-01	-1.517	1.05E-01	-1.020	2.14E-01	-1.241	1.95E-01	-1.258	1.90E-01	-1.258	1.90E-01
PC2	-3.124	1.93E-03	-2.156	8.73E-02	-2.927	3.36E-03	-1.204	1.54E-01	-1.185	1.61E-01	-1.185	1.61E-01
PC3	-0.560	5.36E-01	0.365	7.39E-01	-0.630	4.81E-01	0.965	2.78E-01	0.957	2.82E-01	0.957	2.82E-01
PC4	1.821	1.73E-02	0.989	2.58E-01	1.789	1.80E-02	-0.356	7.20E-01	-0.376	7.05E-01	-0.376	7.05E-01
PC5	0.480	5.35E-01	0.424	6.19E-01	0.627	4.14E-01	0.451	6.33E-01	0.468	6.21E-01	0.468	6.21E-01
Peanut Allergy	-	-	-	-	0.237	9.87E-03	-	-	-	-	-0.506	4.31E-01
HLA-DQA1*01:02	-0.071	3.40E-01	-0.115	1.66E-01	-0.095	2.03E-01	**0.342**	**2.21E-04**	**0.339**	**2.58E-04**	**0.339**	**2.58E-04**

Table S3: Association analysis results for HLA alleles with primary phenotypes of peanut allergy, psIgG4 and psIgE at 60 months in participants of self-reported European ethnicity. Results with p<0.05 are bolded.

**Alleles**	**Allele Frequency (%)**			**PA**		**psIgE**				**psIgG4**			
	**Full Group**	**Avoiders**	**Consumers**	**Avoiders (N=211)**		**Avoiders (N=211)**		**Consumers (N=191)**		**Avoiders (N=211)**		**Consumers (N=191)**	
	**(N=402)**	**(N=211)**	**(N=191)**	**OR**	**P-Value**	**Beta**	**P-Value**	**Beta**	**P-Value**	**Beta**	**P-Value**	**Beta**	**P-Value**
HLA-A*02:01	30.71%	31.91%	29.40%	0.55	1.65E-01	-0.228	1.50E-01	0.187	1.13E-01	**-0.165**	**2.99E-02**	0.084	4.11E-01
HLA-B*07:02	11.68%	12.63%	10.66%	1.06	9.02E-01	-0.190	3.17E-01	-0.121	4.09E-01	0.034	7.09E-01	0.196	1.19E-01
HLA-C*07:02	11.23%	11.36%	11.08%	1.39	5.12E-01	-0.208	2.91E-01	-0.160	2.78E-01	-0.005	9.60E-01	0.161	2.03E-01
HLA-G*01:01	83.29%	84.50%	81.94%	1.00	4.58E-01	-0.211	6.54E-01	0.234	4.25E-01	**-0.891**	**1.00E-04**	-0.416	1.07E-01
HLA-DQA1*01:02	14.95%	15.76%	14.05%	**3.54**	3.63E-03	0.012	9.44E-01	0.023	8.64E-01	-0.060	4.70E-01	**0.395**	**3.34E-04**
HLA-DQA1*05:01	13.53%	14.29%	12.70%	1.81	1.90E-01	0.147	4.19E-01	0.020	8.87E-01	0.041	6.44E-01	-0.172	1.58E-01
HLA-DQA1*01:03	6.06%	5.91%	6.22%	0.59	5.00E-01	0.152	5.39E-01	0.181	3.07E-01	0.211	8.05E-02	**0.382**	**1.07E-02**
HLA-DQB1*03:02	12.72%	10.29%	15.41%	0.82	7.08E-01	-0.202	3.17E-01	-0.234	6.42E-02	-0.182	6.59E-02	-0.154	1.55E-01
HLA-DQB1*02:01	12.72%	13.73%	11.62%	1.73	2.21E-01	0.165	3.77E-01	-0.025	8.63E-01	0.046	6.15E-01	-0.208	9.03E-02
HLA-DQB1*06:02	10.54%	12.01%	8.92%	3.02	1.14E-02	-0.076	6.84E-01	-0.038	7.96E-01	-0.032	7.27E-01	**0.333**	**8.32E-03**

Table S4: Association of HLA haplotypes with frequency >=5% with primary phenotypes of peanut allergy, psIgG4 and psIgE at 60 months. Results with p<0.05 are bolded.

**Haplotypes**	**Haplotype Freq (%)**			**PA**		**psIgE**				**psIgG4**			
	**Full Group**	**Avoiders**	**Consumers**	**Avoiders**		**Avoiders**		**Consumers**		**Avoiders**		**Consumers**	
				**OR**	**P-Value**	**Beta**	**P-Value**	**Beta**	**P-Value**	**Beta**	**P-Value**	**Beta**	**P-Value**
**HLA-DQA1~HLA-DQB1**													
DQA1*05:01~DQB1*02:01	12.00%	12.84%	11.13%	1.28	5.26E-01	0.143	3.92E-01	-0.143	3.14E-01	0.036	6.73E-01	**-0.305**	**4.31E-03**
DQA1*05:05~DQB1*03:01	11.51%	8.95%	14.17%	0.46	1.75E-01	-0.005	9.81E-01	0.117	3.60E-01	0.098	3.20E-01	0.021	8.33E-01
DQA1*01:02~DQB1*06:02	11.41%	13.62%	9.11%	1.96	6.35E-02	-0.089	5.89E-01	-0.054	7.10E-01	-0.036	6.67E-01	**0.242**	**2.69E-02**
DQA1*02:01~DQB1*02:02	11.21%	12.84%	9.51%	1.06	8.79E-01	0.276	1.02E-01	0.167	2.57E-01	0.113	1.84E-01	-0.137	2.22E-01
DQA1*01:01~DQB1*05:01	10.02%	9.73%	10.32%	0.58	2.96E-01	-0.101	5.85E-01	0.046	7.48E-01	-0.023	8.09E-01	-0.019	8.61E-01
DQA1*03:01~DQB1*03:02	9.33%	7.78%	10.93%	0.96	9.32E-01	-0.160	4.33E-01	-0.213	1.21E-01	-0.169	1.02E-01	-0.056	5.91E-01
DQA1*03:03~DQB1*03:01	6.35%	6.03%	6.68%	1.28	6.23E-01	0.272	2.14E-01	0.088	6.11E-01	0.009	9.32E-01	-0.055	6.79E-01
**HLA-DQA1~HLA-DQB1~HLA-DRB1**													
DQA1*05:01~DQB1*02:01 ~DRB1*03:01	12.67%	14.44%	10.80%	0.93	8.70E-01	-0.017	9.31E-01	0.064	7.05E-01	-0.021	8.35E-01	**-0.262**	**4.73E-02**
DQA1*02:01~DQB1*02:02 ~DRB1*07:01	12.40%	14.17%	10.51%	0.92	8.63E-01	0.268	1.71E-01	0.145	3.77E-01	0.164	1.07E-01	-0.223	8.21E-02
DQA1*01:02~DQB1*06:02 ~DRB1*15:01	10.06%	11.76%	8.24%	2.16	8.23E-02	-0.173	3.91E-01	-0.018	9.18E-01	-0.028	7.86E-01	**0.409**	**2.94E-03**
DQA1*01:01~DQB1*05:01 ~DRB1*01:01	9.09%	7.75%	10.51%	0.86	7.99E-01	-0.132	5.78E-01	-0.106	5.21E-01	-0.087	4.80E-01	-0.039	7.65E-01
DQA1*03:03~DQB1*03:01 ~DRB1*04:01	6.20%	6.42%	5.97%	1.99	2.05E-01	0.410	1.05E-01	0.030	8.84E-01	0.047	7.20E-01	-0.026	8.74E-01

Table S5. Association of HLA Class II gene expression in CD4 T cells with rs17612852 and DQA1*01:02 genotypes. An additive model was used for rs17612852 SNP genotype, and a dominant model was used for HLA-DQA1*01:02 carrier status.

Gene	Variant	Minor allele	Beta*	P-value*
DRA	rs17612852	G	0.393	2.36E-02
	DQA1*01:02	carrier	0.388	9.78E-02
DRB5	rs17612852	G	0.466	3.79E-02
	DQA1*01:02	carrier	0.821	4.53E-03
DRB1	rs17612852	G	0.414	3.66E-03
	DQA1*01:02	carrier	0.534	5.00E-03
DQA1	rs17612852	G	1.303	1.66E-07
	DQA1*01:02	carrier	1.182	7.72E-04
DQB1	rs17612852	G	1.567	8.34E-13
	DQA1*01:02	carrier	1.396	1.27E-05
DPA1	rs17612852	G	0.418	7.08E-03
	DQA1*01:02	carrier	0.457	3.30E-02
DPB1	rs17612852	G	-0.035	7.90E-01
	DQA1*01:02	carrier	-0.007	9.68E-01

**Linear regression adjusted for sex, race, allergic status, treatment group, and cell viability.*

Table S6: Sample sizes for each phenotype by timepoint from the total of 267 Consumers and 275 avoiders.

Phenotypes	Consumers	Avoiders	Consumers	Avoiders	Consumers	Avoiders	Consumers	Avoiders	Consumers	Avoiders
	V0		V12		V30		V60		V72	
psIgE	265	274	262	273	260	264	255	266	214	238
Ara h 1 IgE	260	271	260	273	259	263	251	261	209	231
Ara h 2 IgE	260	271	260	273	259	263	251	261	209	230
Ara h 3 IgE	260	271	260	273	259	263	251	261	209	230
Ara h 8 IgE	260	270	260	273	259	263	250	261	208	230
Ara h 9 IgE	260	271	259	272	258	262	251	261	207	230
psIgG4	260	269	257	273	259	264	254	265	213	238
Ara h 1 IgG4	241	241	231	238	234	231	249	254	152	214
Ara h 2 IgG4	241	241	231	238	234	231	249	254	152	214
Ara h 3 IgG4	241	241	231	238	234	231	249	254	152	214
Ara h 8 IgG4	241	241	231	238	234	231	249	254	152	214
Ara h 9 IgG4	241	241	231	238	234	231	249	254	152	214

Table S7: Linear regression models for psIgG4 to determine the direct vs. indirect effects of *MALT1* (rs57265082) and HLA-DQA1*01:02. Five linear regression models (M1-M5) were run on log10(psIgG4), all of which included covariates of age, sex and 5PCs. In all models rs57265082 and HLA-DQA1*01:02 were coded as dominant (i.e. carrier vs. non-carrier); the minor heterozygote and homozygote for rs57265082 were combined to facilitate the interactions being tested with peanut exposure. Exact variables in each model are listed as Model components in the table. M1 was run in the full group of avoiders and consumers combined, and included both genetic variants, log10(psIgE), peanut consumption and interactions with peanut consumption. Models M2-M3 were performed in the Avoiders alone to see how much of the effect of each genetic variant on psIgG4 was mediated through psIgE. Models M4-M5 were performed in Consumers alone to see how much of the effect of each genetic variant on psIgG4 was mediated through psIgE.

	**FULL GROUP N=542**			**AVOIDERS N=275**						**CONSUMERS N=267**					
	**M1**			**M2**			**M3**			**M4**			**M5**		
**Model components**	**Est**	**P-value**		**Est**	**P-value**		**Est**	**P-value**		**Est**	**P-value**		**Est**	**P-value**	
Age	0.032	2.30E-02	*	0.026	1.99E-01		0.022	2.51E-01		0.024	3.07E-01		0.039	6.20E-02	.
Sex	0.062	2.11E-01		0.059	4.07E-01		0.019	7.75E-01		0.188	2.13E-02	*	0.103	1.61E-01	
PC1	-0.151	8.09E-01		-1.456	8.31E-02	.	-0.520	5.18E-01		-1.556	1.52E-01		0.181	8.56E-01	
PC2	-1.179	4.69E-02	*	-2.585	8.81E-03	**	-2.061	2.69E-02	*	-1.780	4.28E-02	*	-0.740	3.52E-01	
PC3	0.104	8.54E-01		-0.257	7.71E-01		-0.163	8.45E-01		0.652	4.58E-01		0.144	8.55E-01	
PC4	0.734	1.83E-01		1.624	2.99E-02	*	1.450	3.95E-02	*	-0.651	5.08E-01		-0.113	8.98E-01	
PC5	0.949	9.65E-02	.	0.623	4.17E-01		0.678	3.49E-01		1.483	1.51E-01		1.296	1.60E-01	
HLA-DQA1*01:02	-0.036	5.65E-01		-0.076	2.44E-01		-0.040	5.10E-01		0.250	2.64E-03	**	0.244	1.05E-03	**
MALT1 (rs57265082)	0.249	2.72E-02	*	0.420	1.63E-04	***	0.235	3.12E-02	*	-0.313	2.48E-02	*	-0.138	2.76E-01	
Log10(psIgE)	0.180	1.92E-08	***				0.175	3.08E-08	***				0.332	5.50E-13	***
Peanut Consumption	0.754	< 2e-16	***												
**HLA*Peanut Consumption**	**0.283**	**2.44E-03**	**												
**MALT1*group Peanut Consumption**	**-0.414**	**1.00E-02**	*												
**psIgE*group Peanut Consumption**	**0.149**	**3.15E-03**	**												

*## Signif. codes: '***' for < 0.001, '**' for < 0.01, '*' for < 0.05, and '.' for < 0.1*
Interaction terms with Peanut Consumption are in bold
Est == Beta estimates of the individual predictor variable in the linear regression models
P-values == p-value of individual predictor variables

Table S8A: Model parameter estimates from the mixed linear model in Fig 3A.

Outcome	Term	Estimate	P-value	95% Lower	95% Upper
Peanut-specific IgG4 (log10)	Intercept	2.02	4e-322	1.97	2.07
	Treatment Group [Peanut Avoidance]	-0.30	3.00E-47	-0.34	-0.26
	Age at Visit (years)	0.14	1.00E-85	0.13	0.15
	(Age at Visit (years))*Treatment Group[Peanut Avoidance]	-0.05	6.00E-14	-0.06	-0.04
	HLA DQA1 01:02:01:01[Carrier]	0.05	8.50E-03	0.01	0.08
	Treatment Group [Peanut Avoidance]*HLA DQA1 01:02:01:01[Carrier]	-0.06	2.30E-03	-0.09	-0.02
Ara h 1 IgG4 (log10)	Intercept	1.60	4e-322	1.57	1.64
	Treatment Group [Peanut Avoidance]	-0.08	5.00E-10	-0.11	-0.06
	Age at Visit (years)	0.03	5.00E-12	0.02	0.04
	(Age at Visit (years))*Treatment Group[Peanut Avoidance]	-0.02	5.80E-07	-0.03	-0.01
	HLA DQA1 01:02:01:01[Carrier]	0.00	7.90E-01	-0.02	0.03
	Treatment Group [Peanut Avoidance]*HLA DQA1 01:02:01:01[Carrier]	0.00	9.10E-01	-0.02	0.03
Ara h 2 IgG4 (log10)	Intercept	1.64	4e-311	1.60	1.68
	Treatment Group [Peanut Avoidance]	-0.22	3.00E-33	-0.26	-0.19
	Age at Visit (years)	0.07	4.00E-30	0.06	0.08
	(Age at Visit (years))*Treatment Group[Peanut Avoidance]	-0.05	4.00E-20	-0.06	-0.04
	HLA DQA1 01:02:01:01[Carrier]	0.08	2.40E-06	0.05	0.11
	Treatment Group [Peanut Avoidance]*HLA DQA1 01:02:01:01[Carrier]	-0.07	2.70E-05	-0.11	-0.04
Ara h 3 IgG4 (log10)	Intercept	2.03	4e-322	1.98	2.07
	Treatment Group [Peanut Avoidance]	-0.18	3.00E-22	-0.21	-0.15
	Age at Visit (years)	0.08	2.00E-35	0.07	0.09
	(Age at Visit (years))*Treatment Group[Peanut Avoidance]	-0.03	6.20E-06	-0.04	-0.02
	HLA DQA1 01:02:01:01[Carrier]	0.01	4.10E-01	-0.02	0.05
	Treatment Group [Peanut Avoidance]*HLA DQA1 01:02:01:01[Carrier]	0.00	8.40E-01	-0.04	0.03
Ara h 8 IgG4 (log10)	Intercept	1.54	4e-322	1.51	1.56
	Treatment Group [Peanut Avoidance]	0.01	2.40E-01	-0.01	0.02
	Age at Visit (years)	0.02	5.40E-08	0.01	0.02
	(Age at Visit (years))*Treatment Group[Peanut Avoidance]	0.00	1.80E-01	0.00	0.01
	HLA DQA1 01:02:01:01[Carrier]	0.00	7.90E-01	-0.01	0.02
	Treatment Group [Peanut Avoidance]*HLA DQA1 01:02:01:01[Carrier]	0.01	2.30E-01	-0.01	0.02
Ara h 9 IgG4 (log10)	Intercept	1.54	4e-322	1.50	1.57
	Treatment Group [Peanut Avoidance]	-0.02	6.50E-02	-0.05	0.00
	Age at Visit (years)	0.04	9.00E-16	0.03	0.05
	(Age at Visit (years))*Treatment Group[Peanut Avoidance]	-0.01	7.60E-02	-0.02	0.00
	HLA DQA1 01:02:01:01[Carrier]	0.03	1.70E-02	0.01	0.06
	Treatment Group [Peanut Avoidance]*HLA DQA1 01:02:01:01[Carrier]	-0.02	8.70E-02	-0.05	0.00

Table S8B: Model parameter estimates from the mixed linear model in Fig 3B.

Outcome	Term	Estimate	P-value	95% Lower	95% Upper
Peanut-Specific IgE (kU/liter,log10)	Intercept	-0.97	8.57E-56	-1.08	-0.86
	Treatment Group [Peanut Avoidance]	0.13	1.33E-02	0.03	0.24
	MALT1 Risk Groups (ALL)[Carrier]	0.26	1.35E-06	0.16	0.37
	Treatment Group [Peanut Avoidance]*MALT1 Risk Groups (ALL)[Carrier]	0.19	4.55E-04	0.08	0.29
	Age at Visit (years)	0.06	2.42E-14	0.04	0.07
	(Age at Visit (years))*Treatment Group[Peanut Avoidance]	-0.01	4.10E-01	-0.02	0.01
Ara h1–Specific IgE (kU/liter, log10)	Intercept	-1.47	2.86E-170	-1.55	-1.40
	Treatment Group [Peanut Avoidance]	0.11	4.73E-03	0.03	0.18
	MALT1 Risk Groups (ALL)[Carrier]	0.19	6.92E-07	0.12	0.26
	Treatment Group [Peanut Avoidance]*MALT1 Risk Groups (ALL)[Carrier]	0.12	2.32E-03	0.04	0.19
	Age at Visit (years)	-0.01	4.16E-02	-0.02	0.00
	(Age at Visit (years))*Treatment Group[Peanut Avoidance]	0.01	5.02E-03	0.00	0.02
Ara h2–Specific IgE (kU/liter, log10)	Intercept	-1.47	5.42E-180	-1.55	-1.39
	Treatment Group [Peanut Avoidance]	0.11	2.79E-03	0.04	0.18
	MALT1 Risk Groups (ALL)[Carrier]	0.20	3.01E-08	0.13	0.27
	Treatment Group [Peanut Avoidance]*MALT1 Risk Groups (ALL)[Carrier]	0.14	1.37E-04	0.07	0.21
	Age at Visit (years)	0.02	8.99E-03	0.00	0.03
	(Age at Visit (years))*Treatment Group[Peanut Avoidance]	0.02	5.63E-03	0.01	0.03
Ara h3–Specific IgE (kU/liter, log10)	Intercept	-1.58	9.95E-212	-1.65	-1.51
	Treatment Group [Peanut Avoidance]	0.05	1.69E-01	-0.02	0.11
	MALT1 Risk Groups (ALL)[Carrier]	0.13	1.24E-04	0.06	0.19
	Treatment Group [Peanut Avoidance]*MALT1 Risk Groups (ALL)[Carrier]	0.11	1.33E-03	0.04	0.17
	Age at Visit (years)	0.01	4.80E-02	0.00	0.02
	(Age at Visit (years))*Treatment Group[Peanut Avoidance]	0.00	7.09E-01	-0.01	0.01
Ara h8–Specific IgE (kU/liter, log10)	Intercept	-1.97	2.978e-320	-2.03	-1.90
	Treatment Group [Peanut Avoidance]	0.04	1.90E-01	-0.02	0.09
	MALT1 Risk Groups (ALL)[Carrier]	0.06	2.31E-02	0.01	0.11
	Treatment Group [Peanut Avoidance]*MALT1 Risk Groups (ALL)[Carrier]	0.08	2.90E-03	0.03	0.13
	Age at Visit (years)	0.09	1.47E-30	0.07	0.10
	(Age at Visit (years))*Treatment Group[Peanut Avoidance]	-0.01	5.40E-02	-0.03	0.00
Ara h9–Specific IgE (kU/liter, log10)	Intercept	-1.86	4.2e-322	-1.90	-1.81
	Treatment Group [Peanut Avoidance]	0.01	6.48E-01	-0.03	0.05
	MALT1 Risk Groups (ALL)[Carrier]	0.08	1.32E-04	0.04	0.12
	Treatment Group [Peanut Avoidance]*MALT1 Risk Groups (ALL)[Carrier]	0.03	2.18E-01	-0.02	0.07
	Age at Visit (years)	0.02	9.08E-12	0.02	0.03
	(Age at Visit (years))*Treatment Group[Peanut Avoidance]	-0.01	8.90E-02	-0.01	0.00

Table S9: Demographics and clinical outcomes of participants included in epitope analysis.

**Demographics**	**Consumers (N=161)**	**Avoiders (N=162)**
Age at screening (mo), mean (SD)	7.61 (1.78)	7.78 (1.75)
Male sex (N)	52.8% (85)	65.4% (106)
Ethnicity (N)		
White	67% (108)	74% (120)
Black	9% (15)	10% (17)
Mixed	16% (26)	12% (19)
Asian	6% (9)	2% (4)
Chinese, Middle Eastern or other	2% (3)	1% (2)
**Outcomes at 60 months**		
PA (allergics/non-allergics)	0/161	37/125
psIgE	0.17 [0.13;0.24]	0.23 [0.15;0.34]
Ara h 1 IgE	0.02 [0.02;0.02]	0.04 [0.02;0.05]
Ara h 2 IgE	0.02 [0.02;0.03]	0.05 [0.03;0.07]
Ara h 3 IgE	0.03 [0.02;0.04]	0.03 [0.02;0.04]
Ara h 8 IgE	0.04 [0.03;0.06]	0.04 [0.03;0.05]
Ara h 9 IgE	0.02 [0.02;0.03]	0.02 [0.02;0.02]
psIgG4	1140.74 [903.63;1440.08]	207.70 [168.44;256.11]
Ara h 1 IgG4	73.29 [60.62;88.60]	40.55 [37.46;43.89]
Ara h 2 IgG4	179.49 [137.83;233.74]	41.81 [38.26;45.69]
Ara h 3 IgG4	423.33 [338.60;529.25]	145.79 [118.49;179.39]
Ara h 8 IgG4	41.03 [37.34;45.09]	45.16 [39.47;51.66]
Ara h 9 IgG4	69.11 [55.23;86.47]	52.99 [44.75;62.75]

Table S10: HLA associations with psIgG in LEAP consumers at 60 months

	**log10(psIgG4)**		**log10(psIgG)**		**log10(psIgG) adjusted for psIgG4**		**log10(psIgG) adjusted for Ara h2 IgG4**	
	Beta	P-value	Beta	P-value	Beta	P-value	Beta	P-value
**HLA-DQA1*01:02**	0.342	2.21E-04	0.111	3.09E-02	-0.017	0.67	-0.018	0.69
**rs17612852**	0.342	5.80E-07	0.142	1.63E-04	0.018	0.54	0.049	0.15

Table S11: Association of peanut allergy (PA) associated HLA variants from previous studies with PA in LEAP peanut avoidance group and psIgG4 in LEAP peanut consumption group. **[A]** PA associated HLA SNPs, **[B]** PA associated imputed HLA alleles. LD for SNPs was calculated with respect to rs17612852 and for alleles with HLA-DQA1*01:02. Peak variants (rs17612852 and HLA-DQA1*01:02) in the LEAP study are bolded.

**Previous studies**					**LEAP study**							
							**Association with Peanut Allergy in peanut avoidance group (N=275)**		**Association with psIgG4 in peanut consumption group (N=267)**			
		**OR**	**P**	**Gene/Nearest gene**	**Alleles**	**MAF***	**OR**	**P**	**Beta**	**P**	**R-sq**	**D'**
**[A] HLA SNPs**												
Hong et al. (*1*)	rs7192	1.7	5.50E-08	HLA-DRA	T/G	0.35	1.55	5.23E-02	0.179	4.99E-03	0.21	0.62
	rs9275596	1.7	6.80E-10	HLA-DQB1|HLA-DQA2	C/T	0.32	1.55	8.37E-02	0.135	4.04E-02	0.36	0.75
	rs33980016	NA	3.20E-11	HLA-DQB1	-	-	-	-	-	-	-	-
Marenholz et al. (*2*)	rs9273440	0.66	6.60E-07	HLA-DQB1	-	-	-	-	-	-	-	-
Asai et al.(*3*)	rs1049213	NA	1.82E-11	HLA-DQB1	-	-	-	-	-	-	-	-
	rs3134976	2.11	2.15E-10	HLA-DQB1|HLA-DQA2	A/C	0.19	1.57	1.23E-01	0.264	5.46E-04	0.76	0.98
	rs3134994	2.11	2.15E-10	HLA-DQB1|HLA-DQA2	T/C	0.19	1.57	1.23E-01	0.264	5.46E-04	0.76	0.98
	rs3135006	2.1	2.45E-10	HLA-DQB1	T/C	0.20	1.61	1.04E-01	0.285	1.75E-04	0.79	0.98
	rs3134995	2.1	3.17E-10	HLA-DQB1|HLA-DQA2	T/C	0.19	1.67	8.31E-02	0.264	5.46E-04	0.75	0.98
	rs3135190	2.08	4.66E-10	HLA-DQB1|HLA-DQA2	T/G	0.20	1.68	7.71E-02	0.285	1.75E-04	0.78	0.98
	rs1049053	2.08	4.66E-10	HLA-DQB1	C/T	0.20	1.61	1.04E-01	0.297	1.21E-04	0.80	0.99
	rs1049225	2.08	4.66E-10	HLA-DQB1	A/G	0.20	1.61	1.04E-01	0.282	2.22E-04	0.79	0.99
	**rs17612852**	**1.99**	**1.86E-09**	**HLA-DQA1|HLA-DQB1**	**G/A**	**0.23**	**1.30**	**3.27E-01**	**0.342**	**5.80E-07**	**ref**	**ref**
	rs9275596	1.76	1.04E-07	HLA-DQB1|HLA-DQA2	C/T	0.32	1.55	8.37E-02	0.135	4.04E-02	0.36	0.75
	rs1612904	1.76	1.36E-07	HLA-DQB1|HLA-DQA2	C/A	0.32	1.55	8.37E-02	0.135	3.80E-02	0.37	0.76
	rs3135002	1.97	1.61E-07	HLA-DQB1|HLA-DQA2	-	-	-	-	-	-	-	-
	rs7774434	0.58	2.22E-07	HLA-DQB1|HLA-DQA2	C/T	0.41	0.68	1.08E-01	0.128	3.41E-02	0.11	0.73
	rs1049056	0.47	2.75E-07	HLA-DQB1	-	-	-	-	-	-	-	-
	rs4947344	1.71	4.19E-07	HLA-DQB1|HLA-DQA2	T/C	0.26	1.71	4.55E-02	0.159	1.99E-02	0.52	0.78
	rs1794275	0.51	8.22E-07	HLA-DQB1|HLA-DQA2	A/G	0.18	0.73	3.08E-01	0.110	1.49E-01	0.04	0.76
	rs7767167	1.95	1.12E-07	HLA-DQB2|HLA-DOB	G/A	0.11	1.67	1.03E-01	0.130	2.30E-01	0.14	0.61
**[B] HLA Alleles**												
Hong et al. (*1*)	**HLA-DQA1*01:02**	**NA**	**2.70E-08**	**HLA-DQA1**	**-**	**0.17**	**1.99**	**4.37E-02**	**0.342**	**2.21E-04**	**ref**	**ref**
	HLA-DQB1*06	NA	5.40E-09	HLA-DQB1	-	-	-	-	-	-	-	-
Martino et al. (*4*)	HLA-DQB1*06	2.26	1.20E-03	HLA-DQB1	-	-	-	-	-	-	-	-
	HLA-DPB1*03	3.23	4.25E-03	HLA-DPB1	-	-	-	-	-	-	-	-
	HLA-DQA1*02	0.36	1.48E-02	HLA-DQA1	-	-	-	-	-	-	-	-
	HLA-DRB1*15	2.15	1.60E-02	HLA-DRB1	-	-	-	-	-	-	-	-
	HLA-DRB1*07	0.34	1.68E-02	HLA-DRB1	-	-	-	-	-	-	-	-
	HLA-DPB1*03:01	3.23	4.25E-02	HLA-DPB1	-	-	-	-	-	-	-	-
	HLA-DQA1*02:01	0.36	1.48E-02	HLA-DQA1	-	0.14	0.92	8.34E-01	0.127	1.95E-01	0.03	1.00
	HLA-DRB1*07:01	0.34	1.68E-02	HLA-DRB1	-	-	-	-	-	-	-	-
	HLA-DQB1*06:02	2.11	1.72E-02	HLA-DQB1	-	0.11	1.96	6.18E-02	0.250	2.16E-02	0.02	1.00
	**HLA-DQA1*01:02**	**1.81**	**1.75E-02**	**HLA-DQA1**	**-**	**0.17**	**1.99**	**4.37E-02**	**0.342**	**2.21E-04**	**ref**	**ref**
